# RUNX2 cooperates with SREBP1 to rewire cancer metabolism and promote aggressiveness

**DOI:** 10.1186/s13046-025-03549-7

**Published:** 2025-10-31

**Authors:** Emanuele Vitale, Mila Gugnoni, Veronica Manicardi, Silvia Muccioli, Federica Torricelli, Benedetta Donati, Simonetta Piana, Gloria Manzotti, Elisa Salviato, Francesca Reggiani, Cristian Ascione, Rebecca Vezzani, Moira Ragazzi, Mattia Forcato, Oriana Romano, Silvio Bicciato, Aaron R. Goldman, Marco Tigano, Alessia Ciarrocchi

**Affiliations:** 1 Laboratory of Translational Research, Azienda USL-IRCCS di Reggio Emilia, Viale Risorgimento 80, Reggio Emilia, 42124 Italy; 2 Clinical and Experimental Medicine PhD Program, University of Modena and Reggio Emilia, Modena, Italy; 3 Pathology Unit, Azienda USL-IRCCS di Reggio Emilia, Reggio Emilia, Italy; 4 Department of Medical and Surgical Sciences for Children and Adults, University of Modena and Reggio Emilia, Modena, Italy; 5 Department of Molecular Medicine, University of Padova, Padova, Italy; 6 Molecular and Cellular Oncogenesis Program, The Wistar Institute, Philadelphia, PA 19104 USA; 7 Department of Pathology and Genomic Medicine, Thomas Jefferson University, 1020 Locust Street, Jefferson Alumni Hall 19107, Philadelphia, PA) USA

**Keywords:** Embryonic transcription factors, Oncogenic gene expression programs, Cancer cell metabolic rewiring, Lipid biosynthesis

## Abstract

Embryonic Transcription Factors (TFs) are often reactivated in cancer, driving developmental gene programs that support phenotypic plasticity. Metabolic adaptation fuels this plasticity by supplying energy and molecular building blocks for growth. RUNX2, the master regulator of bone morphogenesis, is ectopically expressed in epithelial cancer, promoting metastasis through trans-differentiation processes like Epithelial-to-Mesenchymal Transition (EMT) and osteomimicry. By combining omics data with functional validation, we demonstrated that RUNX2 drives cancer cell metabolic rewiring by repressing mitochondrial respiration while promoting anabolic processes. We showed that RUNX2 upregulates key genes of lipid biosynthesis by regulating and cooperating with SREBP1. In vivo expression analysis in thyroid and breast cancer patients confirmed that lipid metabolism and *SREBF1* expression are associated with increased metastatic potential and clinical aggressiveness. These findings emphasize the RUNX2 role in cancer plasticity and indicate metabolic adaptation as an integral part of the trans-differentiation program induced by this TF during cancer progression.

**Supplementary Information:**

The online version contains supplementary material available at 10.1186/s13046-025-03549-7.

## Introduction

Cancer is a heterogeneous disease with a high potential for phenotypic adaptation [1–4].

Transcriptional plasticity – guaranteed by the inherent ductility of the epigenome – is a key mechanism behind phenotypic adaptation [5]. Transcription Factors (TFs) govern this plasticity by coordinating multidimensional networks of regulatory elements to synchronize and fine-tune the expression of genes that execute the oncogenic program. Oncogenic TFs are essential for cancer cells, providing promising opportunities to develop novel therapeutic interventions [6–8]. Yet, several knowledge gaps regarding how oncogenic TFs regulate their networks still exist, hindering therapeutic advancements.

RUNX2 is an embryonic TF essential for bone development [9–13] and involved in the morphogenesis of other organs [14, 15]. RUNX2 is overexpressed in various cancers, including breast (BC) and thyroid cancer (TC) [16, 17], where it promotes metastasis by regulating key functions such as stress resistance and phenotypic plasticity through processes like the Epithelial-to-Mesenchymal Transition (EMT) [15, 18, 19] and osteomimicry [20–26]. Despite its essential role, a comprehensive description of the transcriptional landscape governed by RUNX2 in cancer and a model that describes how its transcriptional function translates into meaningful biological effects have yet to be established.

By integrating multi-omics profiles with patients’ clinical data, we recently demonstrated that the RUNX2 transcriptional program is organized in discrete modules, each underlining specific biological functions. We also predicted a RUNX2-dependent gene module related to metabolic processes as associated with TC metastatic spreading [27]. The ability to rapidly rewire metabolism is a major feature of metastatic cancer cells [28] that not only ensures energy supply but also provides the building blocks for oncogenic processes and serves to improve stress resistance. In this work, by employing cutting-edge genomic approaches followed by functional validations, we elucidated the genomic functions of RUNX2, shedding light on the mechanisms through which this TF promotes cancer progression. We showed that RUNX2 controls both cancer cell catabolism and anabolism by repressing oxidative phosphorylation (OXPHOS) while promoting lipid biosynthetic pathways. We demonstrated for the first time that RUNX2 directly drives the expression of key genes involved in *de novo* synthesis of fatty acids and cholesterol, including *SREBF1*, which encodes SREBP1, the master regulator of these pathways [29, 30]. We also showed that RUNX2 cooperates with SREBP1 for the regulation of its direct gene program, describing for the first time the functional interplay between these two factors. At last, we confirmed the relevance of RUNX2 transcriptional regulation in vivo in two independent retrospective cohorts of 48 TC and 79 BC patients, showing that *SREBF1* is overexpressed in TCs and BCs that developed distant metastases.

## Results

### Transcriptional landscape of RUNX2 in TCs

**Fig. 1:**
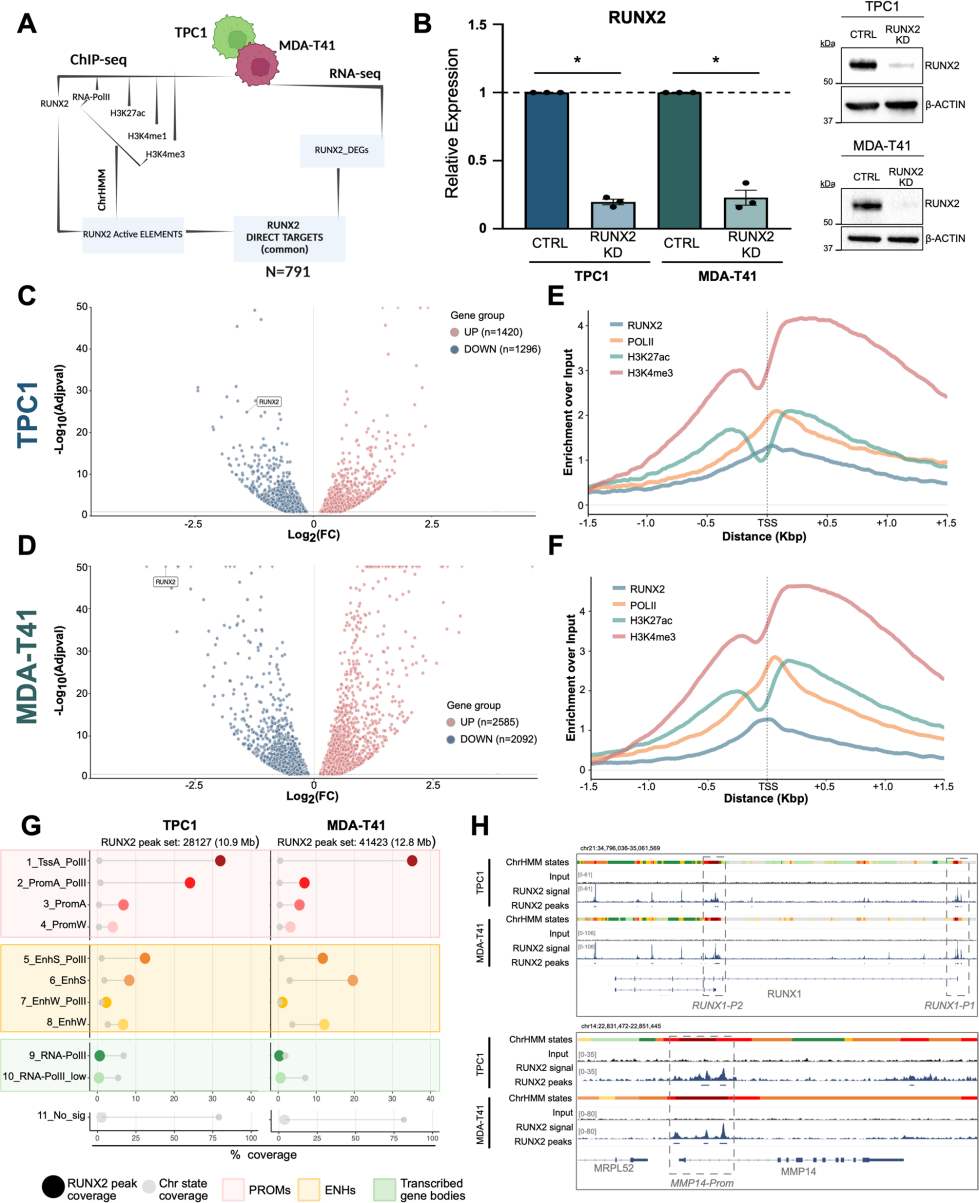
RUNX2 transcriptional landscape. (**A**) Schematic representation of the analysis workflow. (**B**) RUNX2 KD assessment by qRT-PCR (left) and Western Blot (right). Bars represent the Mean ± SEM of RUNX2 fold expression over control (CTRL). *p ≤ 0.05. **C-D**) Volcano plot displaying deregulated genes (DEGs) between RUNX2 KD and CTRL TPC1 (**C**) and MDA-T41 (**D**). Blue and red dots represent significantly (Adj p-value ≤ 0.1) downregulated and upregulated genes, respectively. **E-F**) Metaprofiles showing the relative distribution of RUNX2, RNA-PolII, H3K27ac, and H3K4me3 around the TSSs of RUNX2-DEGs in TPC1 (**E**) and MDA-T41 (**F**). **G**) Lollipop chart showing the overlap between RUNX2-peaks with the 11 chromHMM states (i.e., coverage) over the total length of RUNX2-peak set (large dots), in TPC1 and MDA-T41 cell lines. Small gray dots highlight the genome-wide coverage percentages of chromHMM states. **H**) Representative IGV tracks showing RUNX2-distribution on the regulatory elements of its targets RUNX1 and MMP14. ChromHMM states were represented with the same color code shown in panel G

To define the transcriptional landscape of RUNX2 in TCs, we designed a combined strategy (Fig. 1A). As previously described [27], we used a CRISPR-interference (CRISPRi) system to silence RUNX2 (Fig. 1B) in TPC1 and MDA-T41, models of primary tumor and TC metastasis. Coherently with our previous findings [31] and with its role in supporting aggressive behaviors, RUNX2 silencing reduced MDA-T41 cell motility and invasiveness (Fig. S1A, S1B). We performed RNA-seq comparing RUNX2 KD and control (CTRL) cells (Fig. 1C-D), identifying 1320 deregulated genes (DEGs) common to the two cell lines (Fig. S1C-D). Gene Ontology (GO) enrichment analysis showed many cancer-supporting pathways enriched with these genes, including cell motility, proliferation, extracellular matrix organization, apoptosis, and regulation of cell differentiation (Fig. S1D).

To map the RUNX2 genomic distribution, we conducted ChIP-seq for RUNX2, RNA-PolII, and histone marks (Fig. 1E-F and S1E). We identified 28,127 RUNX2-associated peaks in TPC1 (Fig. 1E; Fig. S1F) and 41,423 in MDA-T41 (Fig. 1F; Fig. S1F). RUNX2 showed a strong enrichment around the TSS, overlapping with RNA-PolII (Fig. 1E, F). To determine the nature and functional status of the RUNX2-bound elements, we integrated the ChIP-seq datasets for RNA-PolII and histone marks using ChromHMM [32]. We defined 11 chromatin states based on the combinations of different chromatin marks (Fig. S1G-H, Table S1). By merging the RUNX2 binding profile with the ChromHMM output, we showed that this TF is enriched on active regulatory elements (96.50% in TPC1 and 95.40% in MDA-MT41) (Fig. 1G). In TPC1, 66.90% RUNX2 peaks were associated with promoter regions, while 29.60% with ENHs, with a prevalence of transcribed strong ENHs (12.30% of total RUNX2-associated elements). RUNX2 showed a different distribution in MDA-T41, with 44.50% RUNX2 binding sites associated with ENHs and 50.80% with promoters. (Fig. 1G). Figure 1H shows the RUNX2 binding on two representative targets.

### Linking RUNX2 to its biological function

**Fig. 2:**
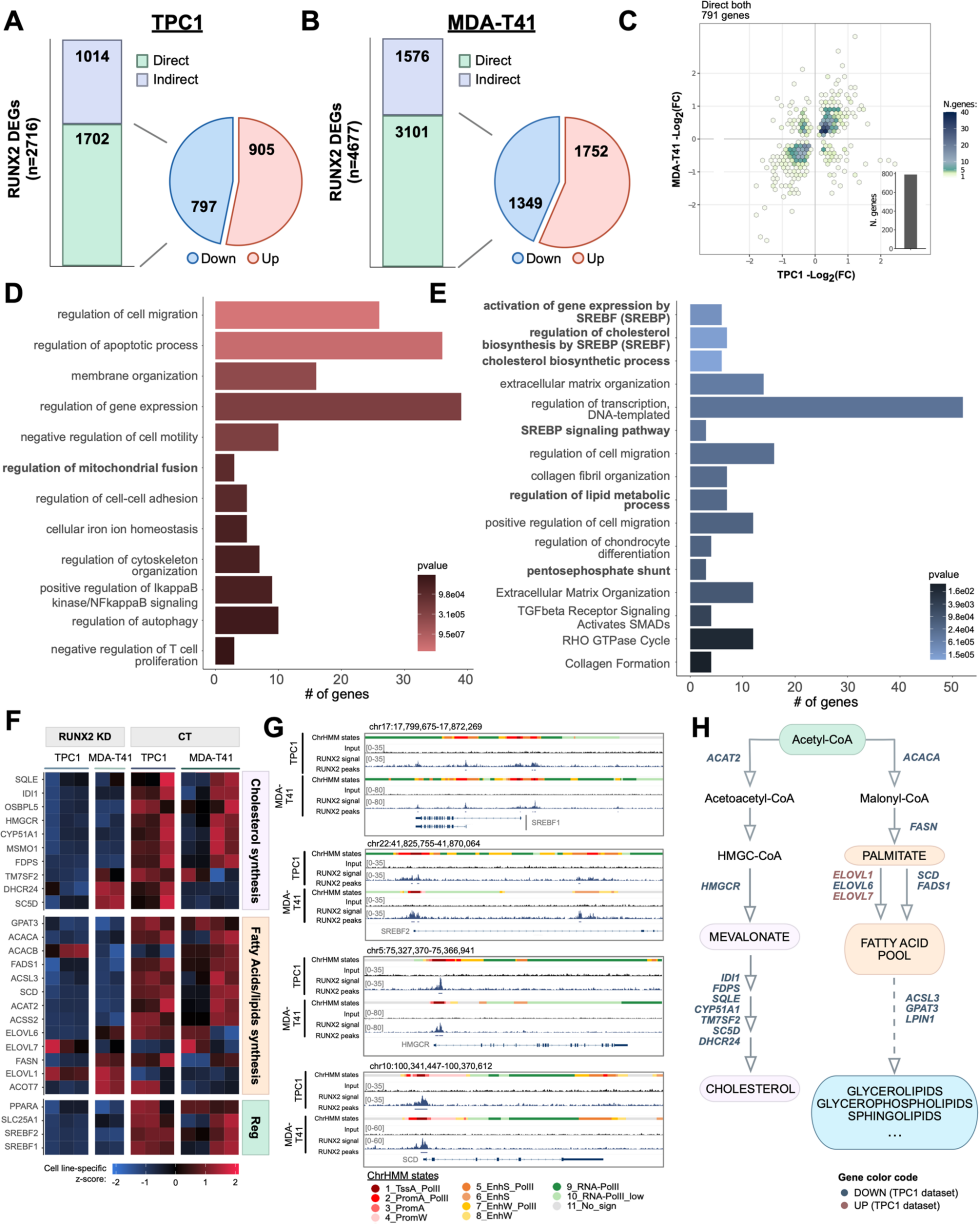
RUNX2 controls lipid metabolism. **A-B**) Distribution of RUNX2 direct and indirect target genes in TPC1 (**A**) and MDA-T41 (**B**). Pie charts show the percentage of downregulated and upregulated genes among the RUNX2-direct targets. **C**) Hexagonal heatmap representing the log2FC of RUNX2-target genes common to TPC1 and MDA-T41 (*n* = 791). Each hexagon represents a different number of genes specified by the fill color scale. 649/791 genes were found to be coherently upregulated or downregulated in the two cell lines. **D**) GO BP and Reactome Pathway enrichment analysis of TPC1 and MDA-T41 common upregulated RUNX2-direct targets. **E**) GO-BP and Reactome Pathway enrichment analysis of TPC1 and MDA-T41 common downregulated RUNX2-direct targets. In bold are enriched terms related to metabolism. **F**) Expression heatmap of selected RUNX2-direct targets involved in lipogenic pathways. **G**) Representative IGV tracks showing RUNX2-distribution on the regulatory elements of *SREBF1*, *SREBF2*, *HMGCR*, and *SCD*. **H**) Graphical representation of cholesterol and fatty acid synthesis pathways, with representative enzymes identified as RUNX2-direct targets (TPC1 dataset). Upregulated genes are marked in red, while the downregulated ones are indicated in blue

To identify the gene program directly regulated by RUNX2, we combined ChIP-seq and RNA-seq datasets. RUNX2 ChIP-peaks were refined by retaining active elements marked with H3K27ac and RNA-PolII and assigned to the nearest TSSs to predict putative target genes. Following multi-omics integration, RUNX2 DEGs (RNA-seq) were classified as direct if they were also ChIP-seq targets or indirect if they were not. With this approach, we identified 1702 genes (62.67%) in TPC1 and 3101 (66.30%) in MDA-T41 as RUNX2 direct targets (Fig. 2A-B), indicating that the global effect of RUNX2 on gene expression is mainly attributable to its direct transcriptional activity rather than to secondary effects. Of these lists, 649 genes displayed coherent changes in both cell lines of which 371 upregulated and 278 downregulated (Fig. 2C). Enrichment analyses of upregulated genes identified cell migration, adhesion, and apoptosis as enriched terms (Fig. 2D), while downregulated genes were enriched for extracellular matrix organization, chondrocyte differentiation, and TGFβ signaling (Fig. 2E). Notably, by analyzing downregulated genes we observed a significant enrichment of processes associated with cholesterol synthesis and *de novo* lipogenesis (Fig. 2E). Many key components of these pathways, including *SCD*, *HMGCR*, *SREBF1*, and *SREBF2*, were downregulated upon RUNX2 KD in TC (Fig. 2F, G). Additionally, *G6PD*, *TKT*, and *H6PD*, key enzymes of the pentose phosphate shunt, which sustains lipid anabolism, and the glucose transporters *SLC2A3*, *SLC2A11*, *SLC2A12* were identified as RUNX2 direct targets in both cell lines (Fig. S2A-C). Finally, upregulated targets include genes influencing mitochondria (Fig. 2D), organelles crucial for cell metabolism and involved in both lipid synthesis and catabolism.

### RUNX2 activity confers a Warburg phenotype to TC cells

**Fig. 3:**
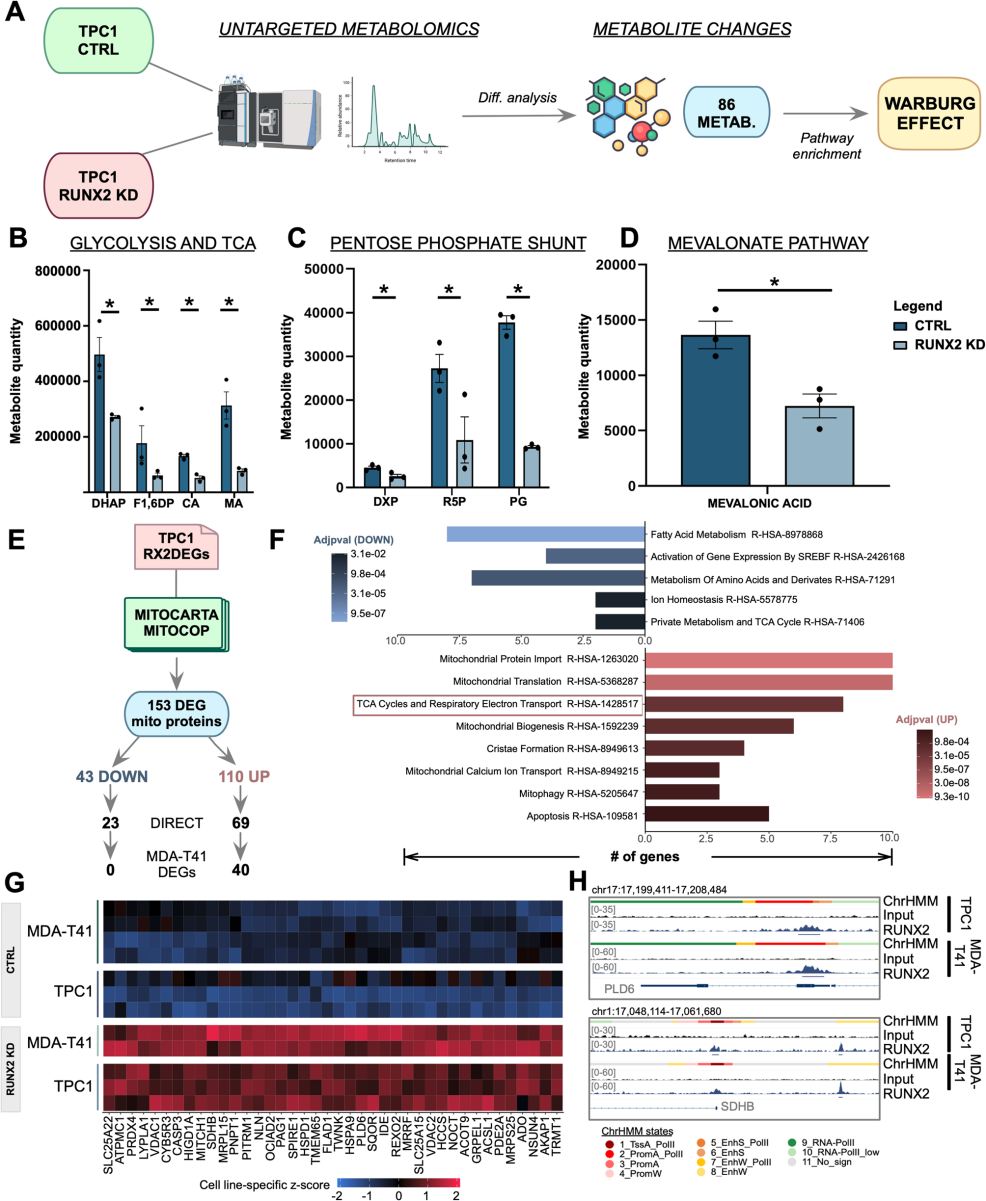
RUNX2 affects metabolic profiles and reduces oxygen consumption. **A**) Schematic workflow of metabolomics analysis conducted on TPC1 upon RUNX2 KD. Differential analysis conducted by comparing RUNX2 KD and CTRL samples identified 86 altered metabolites. **B-D**) Histograms showing the concentration of metabolites belonging to the tricarboxylic acid cycle (TCA) and glycolysis (**B**), pentose-phosphate (**C**), and mevalonate pathway of cholesterol synthesis (**D**) that we identified as significantly (*) downregulated upon RUNX2 silencing. **E**) Workflow of the strategy used to identify mitochondrial proteins affected by the RUNX2 loss. **F**) Reactome Pathway enrichment analysis on down (blue bars) and upregulated (red bars) mitochondrial protein genes. **G**) Expression heatmap of RUNX2-directly regulated mitochondrial protein common to TPC1 and MDA-T41. **H**) Representative IGV tracks showing RUNX2-distribution on the regulatory elements of its targets *PLD6* and *SDHB*

To validate these transcriptomic data, we performed untargeted metabolomics in TPC1 cells. The intracellular concentrations of 86 metabolites were significantly affected by RUNX2 KD (Fig. 3A; Table S2). Pathways enrichment analysis by MetaboAnalyst identified Warburg Effect as the sole statistically significant enriched term (Adj p-value = 0.085), consistent with the observed decrease of Tricarboxylic Acid Cycle (TCA) and glycolysis intermediates (Fig. 3B). Metabolites of the pentose-phosphate pathway were also underrepresented in RUNX2 KD cells (Fig. 3C), coherently with the downregulated expression of *G6PD*, *TKT*, and *H6PD* (Fig. S2A). Moreover, 18 lipids showed increased concentration (Table S2). Finally, the intracellular level of mevalonic acid was significantly decreased in RUNX2 KD cells (Fig. 3D) in line with the decreased expression of *HMGCR* (Fig. 2F), the rate-limiting step enzyme of the mevalonate pathway (Fig. 2H).

The results of untargeted metabolomics from TPC1 were confirmed in MDA-T41, in which 43 metabolites showed an altered profile upon RUNX2 silencing with the Warburg Effect predicted as a significantly enriched pathway (Fig. S3A; Table S3). The two models of TC shared 22 altered metabolites (Fig. S3B, Table S3). No lipids were detected as differentially represented in MDA-T41 in this analysis, but an increase in oleic acid was observed (Table S3).

### RUNX2 loss affects mitochondrial function in TCs

**Fig. 4:**
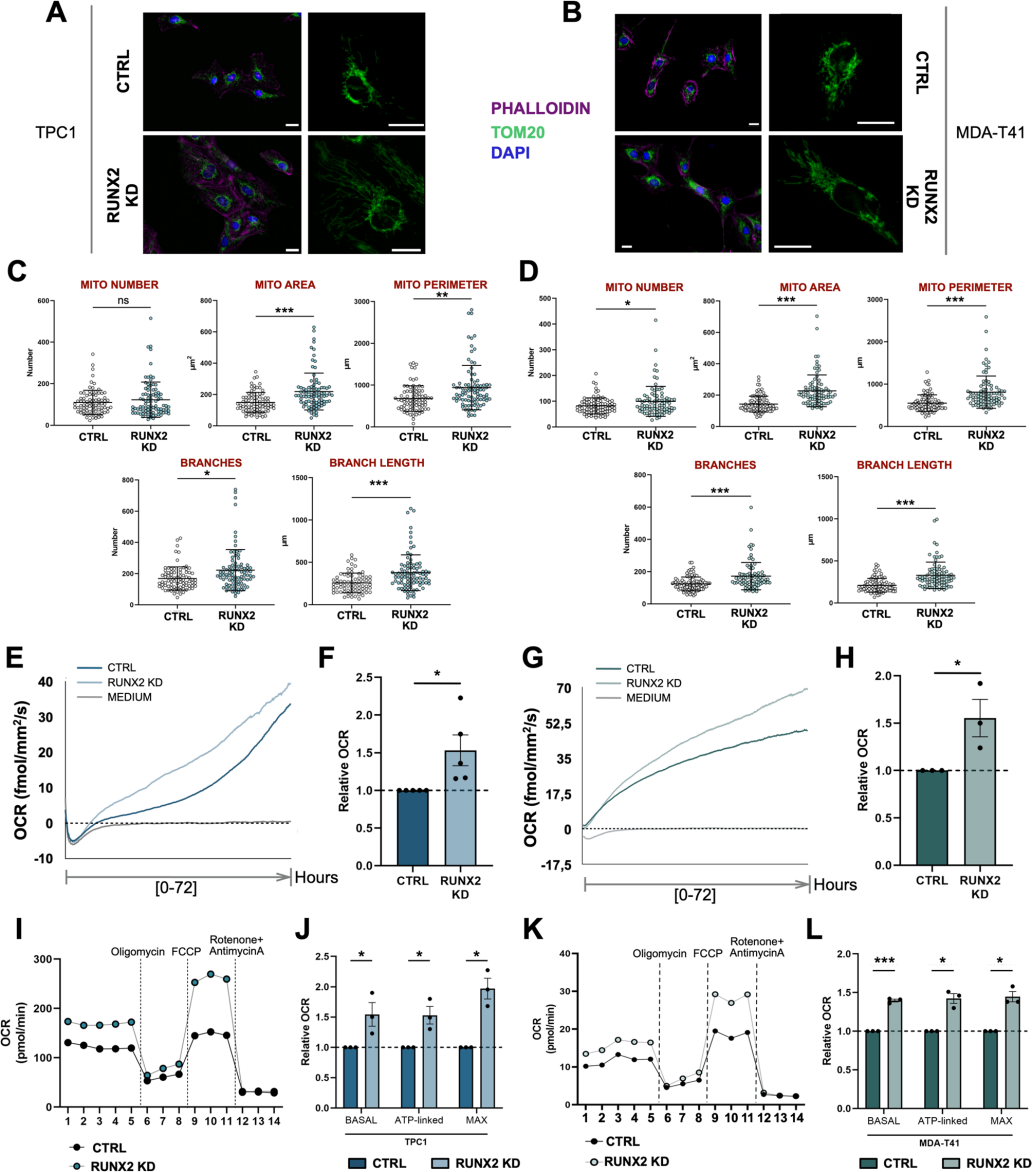
RUNX2 controls mitochondrial organization and represses OXPHOS. **A-B**) TOM20 (green) and phalloidin (magenta) staining on RUNX2 KD and CTRL cells in TPC1 (**A**) and MDA-T41 (**B**). **C-D**) Mitochondria number, area, perimeter, branches, and branch length quantification by the Mitochondria-Analyzer ImageJ Plug-In in TPC1 (**C**) and MDA-T41 (**D**). Graphs showed the Mean ± St.Dev of each parameter in CTRL and RUNX2 KD samples. Experiments were conducted in triplicate and at least 25 cells/replicate were considered for the quantification analyses. **E-H**) Resipher live OCR measurement in TPC1 (**E-F**) and MDA-T41 (**G-H**). Panels E and G show the OCR curves of representative experiments in TPC1 (**E**) and MDA-T41 (**G**). Histograms show the FC increase of the OCR normalized on cell number in TPC1 (**F**) and MDA-T41 (**H**) RUNX2 KD cells compared to CTRL cells. **I-L**) Seahorse Mitostress test analysis in TPC1 (**I**-**J**) and MDA-T41 (**K-L**). Panels I and K show the OCR curves of representative experiments in TPC1 (**I**) and MDA-T41 (**K**). Histograms (**J, L**) show the basal, ATP-production linked, and maximum OCR FC increase in TPC1 (**J**) and MDA-T41 (**L**) RUNX2 KD compared to CTRL cells.*p≤ 0.05. **p≤ 0.001. ***p≤0.0001.

Cancer cells experiencing the Warburg Effect downregulate OXPHOS and promote glycolysis. Mitochondria play a key role in this process. Among the RUNX2 DEGs in TPC1, 153 genes were annotated as mitochondrial proteins according to MitoCarta and MitoCoP databases (Fig. 3E). Several keystone mitochondrial functions – including regulation of protein import in the organelle, translation, cristae biogenesis, calcium signaling, and mitophagy – were upregulated following RUNX2 silencing (Fig. 3F) and “*TCA Cycles and Respiratory Electron Transport*” was among the top-scoring upregulated pathway. Many members of the mitochondrial respiratory chain and ATP-synthase complex were perturbed following RUNX2 KD (Fig. S3C). Forty mitochondrial proteins were identified as upregulated RUNX2 direct targets in both cell lines (Fig. 3G-H). These data indicate a direct role for RUNX2 in influencing mitochondrial function by attenuating the expression of mitochondrial proteins. To provide phenotypic validation, we analyzed morphological parameters of mitochondrial health and functionality. Immunofluorescence (IF) labeling showed a profound reorganization of the mitochondrial network following RUNX2 loss in both TPC1 and MDA-T41 (Fig. 4A-B, Fig. S3D). Compared to CTRL, RUNX2 KD cells showed more elongated mitochondria that occupied a larger area of cytoplasm (Fig. 4C-D). A similar phenotype was observed by live-cell imaging of the mitochondrial network stained by TMRM (Fig. S3E). These changes were consistent in both the cell models and were not accompanied by notable alterations in the number of mitochondria in TPC1 (Fig. 4C) or the amount of mtDNA (Fig. S3F). Mitochondrial elongation is usually indicative of an increased OCR. Real-time live cell oxygen sensing showed a 1.5-fold increase in the OCR (Fig. 4E-H) when normalized to total cell number (Fig. S3G) in both RUNX2 KD TPC1 (Fig. 4E, F) and MDA-T41 (Fig. 4G, H) as compared to control cells. To gain further insights into the OXPHOS capacity of the cells, we performed the Seahorse XF Cell Mito Stress Test in TPC1 and MDA-T41 (Fig. 4I-L). RUNX2 KD was associated with a higher basal, ATP-production linked, and maximal OCR as compared to controls (Fig. 4I-L).

Taken altogether, our data support a model whereby RUNX2 directly represses mitochondrial function to attenuate TC cells OXPHOS.

### RUNX2 controls de novo lipogenesis affecting the lipids profile of TC

**Fig. 5:**
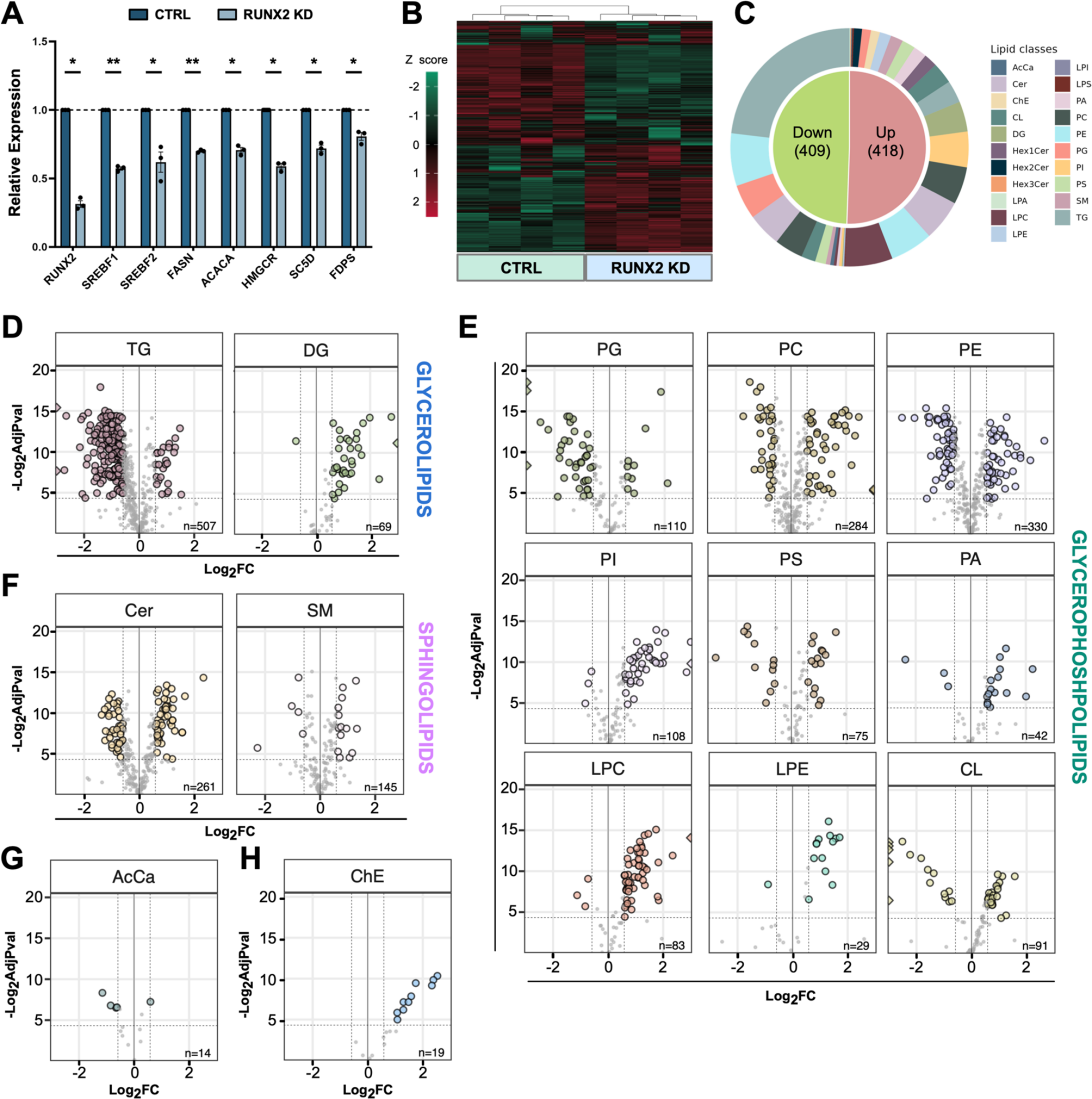
RUNX2 KD produces profound alterations of the lipid profile of TC cells. (**A**) qRT-PCR showing the FC expression of a representative panel of lipogenesis-related genes in RUNX2 KD TPC1 compared to CTRL. Bars represent the Mean ± SEM of three independent experiments. *p ≤ 0.05. **p ≤ 0.001. (**B**) Hierarchical clustering heatmap of lipid species in CTRL and RUNX2 KD TPC1. (**C**) Pie chart indicating the fraction of lipid species displaying an increase (Up) or decrease (Down) in their intracellular concentration upon the RUNX2 loss. The outer ring shows the distribution of the different classes of the altered lipids. **D-H**) Volcano plots showing the altered lipid species belonging to the glycerolipids (**D**), glycerophospholipids (**E**), sphingolipids (**F**), acyl-carnitine (**G**), and cholesterol esters (**H**).

Our data highlighted that RUNX2, besides its function in OXPHOS, directly promotes the expression of key genes of fatty acids and cholesterol synthesis (Fig. 5A, Fig. S4A), suggesting an involvement in cell lipid homeostasis. To validate this function, we performed untargeted lipidomic analysis in CTRL and RUNX2 KD TPC1. In line with the transcriptomic data, this analysis showed a massive reorganization of the cancer cell lipid profile following RUNX2 silencing (Fig. 5B, Fig. S4B). Differential analysis identified significant alterations in 827 lipid species belonging to different lipid classes (Fig. 5C). Triglycerides (TG) displayed the most pronounced perturbation with a marked decrease detected in RUNX2 KD cells (Fig. 5D). Besides, many membrane lipids exhibited alteration upon RUNX2 KD (Fig. 5E-F; Fig.S4C). Among these, Phosphatidylglycerols (PG) showed an overall decrease while Phosphatidic acids (PA), Phosphatidylinositols (PI), Lysophosphatidylcholines (LPC), Lysophosphatidylethanolamines (LPE), and Hexosylceramides (Hex1Cer, Hex2Cer) showed an increase in their levels following RUNX2 silencing (Fig. 5C, E; Fig. S4C). We also observed a significant alteration in Cardiolipins (CLs), which are important components of the mitochondrial inner membrane (Fig. 5E), in Acylcarnitines (AcCA), and Cholesterol Esters (ChE) (Fig. 5G-H).

**Fig. 6:**
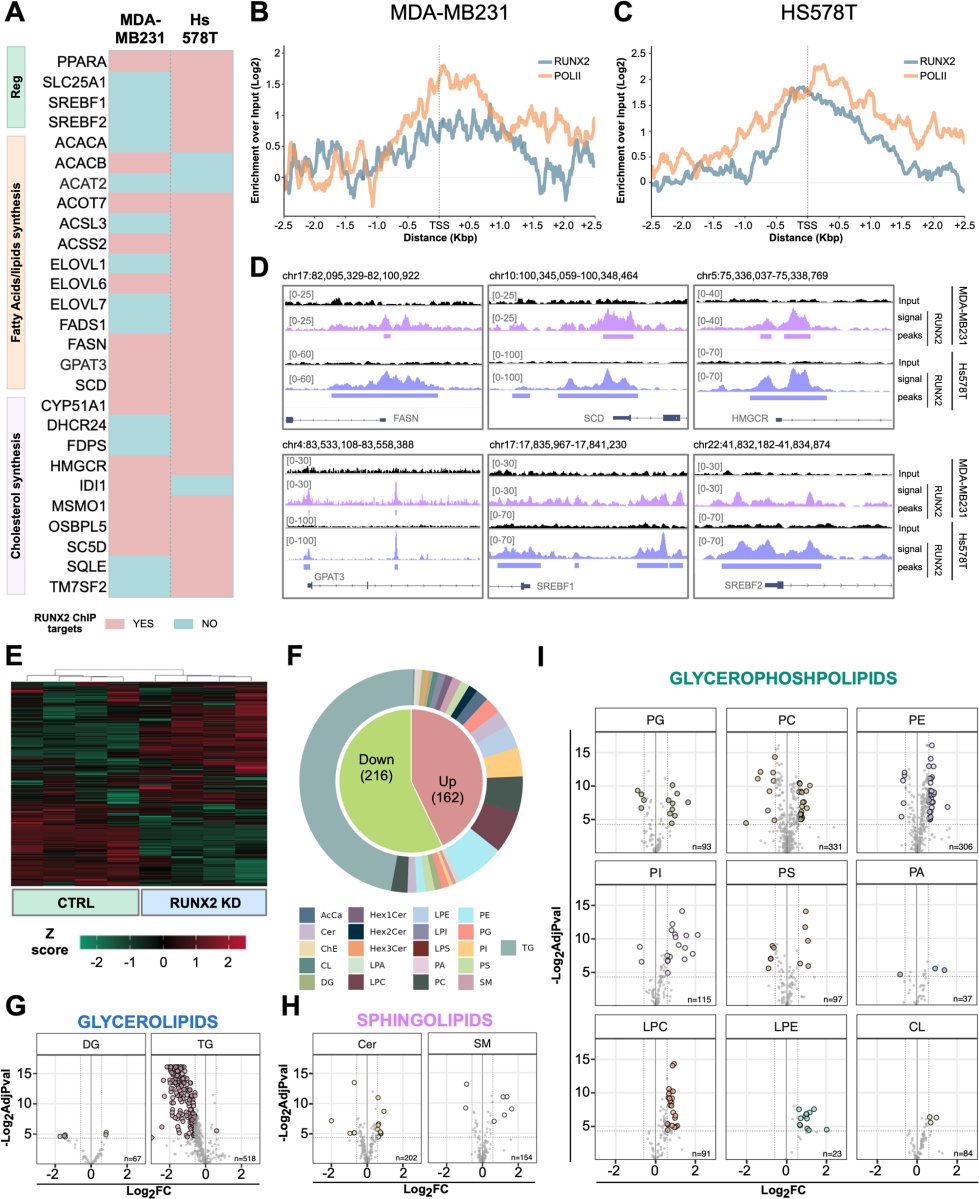
RUNX2 controls lipid metabolism in BC cells. **A**) Graphical representation of metabolism-related RUNX2-ChIP targets confirmed in MDA-MB231 and Hs578T. **B-C**) Metaprofiles showing the relative distribution of RUNX2 and RNA-PolII in a 5 Kbp window around the TSSs of the lipid-metabolism RUNX2-targets confirmed in MDA-MB231 (*N* = 14) (**B**) and Hs578T (*N* = 24) (**C**). **D**) Representative IGV tracks showing RUNX2-distribution on the regulatory elements of *FASN*, *SCD*, *HMGCR*, *GPAT3*,* SREBF1*, and *SREBF2* in MDA-MB231 and Hs578T. **E**) Hierarchical clustering heatmap of lipid species in CTRL and RUNX2 KD MDA-MB231. **F**) Pie chart indicating the fraction of lipid species displaying an increase (Up) or decrease (Down) in their intracellular concentration upon the RUNX2 loss. The outer ring shows the distribution of the different classes of the altered lipids. **G-I**) Volcano plots showing the altered lipid species belonging to the glycerolipids (**G**), sphingolipids (**H**), and glycerophospholipids (**I**).

Lipid metabolism reprogramming confers metastatic potential, contributing to cancer cell migration, seeding, and growth in ectopic sites [33]. To investigate whether the RUNX2 effect on metabolism represents a cross-sectional mechanism in other cancers, we used MDA-MB231 and Hs578T as BC metastasis and primary tumor models. We performed ChIP-seq analysis in these BC cells, confirming RUNX2 binding on the regulatory elements of lipid-synthesis-related genes. Specifically, of the 27 lipid-related targets identified in TC (Fig. 2F), 14 (51.85%) and 24 (88.89%) were confirmed in MDA-MB231 and Hs578T, respectively (Fig. 6A). In both cell models, RUNX2 showed an enrichment around the TSS of these genes, overlapping with RNA-PolII (Fig. 6B-C). Key examples included *HMGCR*, *FASN*, *SCD*, *GPAT3*, *SREBF1*, and *SREBF2* (Fig. 6D).

To provide functional validation, we silenced RUNX2 by CRISPRi in MDA-MB231 cells (Fig. S4D) and analyzed the effect on metabolic rewiring. Real-time live OCR measurement and Seahorse XF Cell Mito Stress Test assays confirmed a significant increase in the respiratory capacity of BC cells upon RUNX2 KD (Fig. S4E-H). To focus on lipid metabolism, we performed untargeted lipidomics. We observed a deep reorganization of the lipidic profile of MDA-MB231 cells as a consequence of RUNX2 KD (Fig. 6E-F), closely mirroring the findings in the TC cell model. TGs confirmed to be the most affected class (Fig. 6G), followed by several classes of structural membrane lipids (Fig. 6H-I).

Taken together, these pieces of evidence demonstrated that RUNX2 transcriptionally sustains metabolic rewiring by simultaneously repressing mitochondrial oxidative respiration and promoting *de novo* lipogenesis in both TC and BC cancer models, highlighting this biological function as a central part of the pro-oncogenic program orchestrated by this TF.

### RUNX2 cooperates with SREBF1 to control genes involved in de Novo lipogenesis

**Fig. 7:**
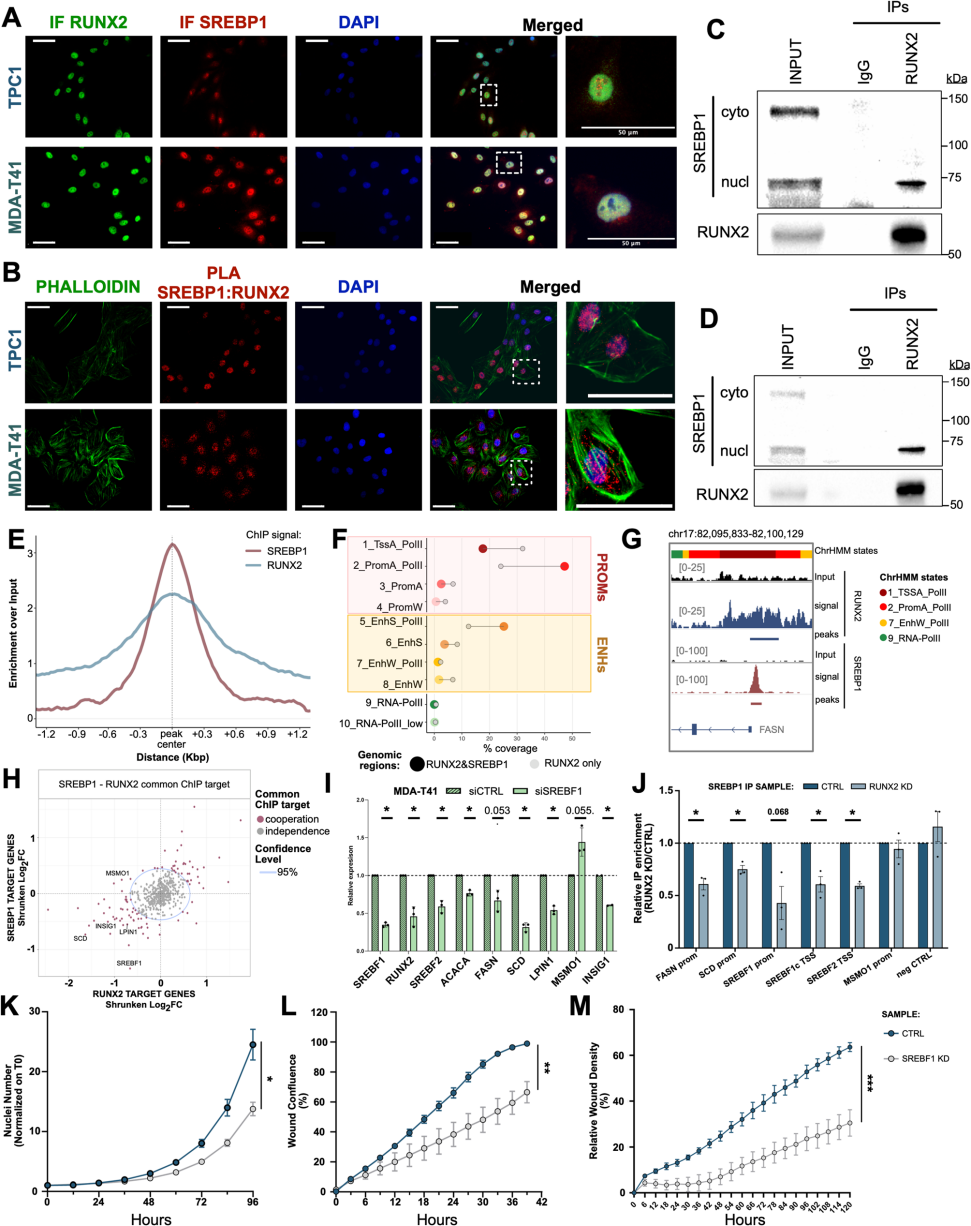
RUNX2 cooperates with SREBP1 to control de novo lipogenesis genes. **A**) Immunofluorescence analysis showing the co-localization of RUNX2 (green) and SREBP1 (red) in TPC1 and MDA-T41 nuclei (blue). **B**) Proximity Ligation Assay (PLA, red signal) of RUNX2 and SREBP1 in TPC1 (top) and MDA-T41 (bottom). Phalloidin (green) was used to identify cell boundaries, while nuclei were marked with DAPI (blue). Scale bars = 50 µM. **C-D**) Co-IP experiments in TPC1 (**C**) and MDA-T41 (**D**). For each cell line, western blots show one representative experiment from two independent replicates (n=2). **E**) Metaporfile showing RUNX2 and SREBP1 enrichment in a 2.4 Kbp window around the center of RUNX2-SREBP1 overlapping peaks. **F**) Lollipop chart showing the overlap of RUNX2 and SREBP1 common peaks (large colored dots) with the 11 chromHMM states (i.e., coverage) over the total length of each set of peaks in TPC1 cell line. **G**) Representative IGV tracks showing RUNX2-SREBP1 colocalization on FASN promoter. **H**) Scatter plot representing the shrunken log2FC of differential expression in RUNX2 KD (x-axis) and SREBP1 KD (y-axis). The light-blue circle indicates the 95% confidence region for the null hypothesis of independence of cooperation between the two TFs. Red dots indicate the genes cooperatively regulated by SREBP1 and RUNX2. **I**) qRT-PCR showing the FC expression of a selected panel of lipogenesis-related genes in MDA-T41 transfected with SREBF1 siRNA (siSREBF1) compared to control siRNA (siCTRL). Bars represent the Mean±SEM of three independent experiments. **J**) qRT-PCR of the SREBP1 ChIP conducted on RUNX2 KD and CTRL TPC1. Fold Change enrichment of RUNX2 KD vs CTRL is represented in the graph. Bars show the Mean ± SEM of three independent experiments. **K**) Proliferation curves of SREBF1 KD and CTRL TPC1. Curves show the Mean ± SEM of three independent experiments. Significance was assessed at 96h. **L-M**) Wound healing (**L**) and invasion (**M**) assays showing reduced motility and invasiveness in SREBF1 KD as compared to CTRL TPC1. Curves show the Mean ± SEM of two and three independent experiments for migration and invasion, respectively. Significance was assessed across all time points.*p≤0.05. **p≤0.001. *** *p*≤0.0001.

*De novo* lipogenesis relies on transcriptional regulation to coordinate the expression of lipogenic genes at the time of need. Our analysis identified *SREBF1* (encoding the SREBP1 protein) as a RUNX2 target in both TC (Fig. 2F-G) and BC cells (Fig. 6D, Fig. S4I), and its expression is significantly correlated with RUNX2 expression in TC patients (Fig. S5A). SREBP1 is a transcription factor that plays an essential role in lipogenesis by driving the expression of enzymes of lipid biosynthesis. Considering that RUNX2 controls the expression of many genes involved in this process (Fig. 2E-H), we reasoned that SREBP1 could represent a mediator and a cooperator of RUNX2 in this pathway. Motif prediction by FIMO algorithm analysis confirmed that the Sterol Regulatory Element (SRE) was enriched in RUNX2 binding sites associated with lipogenic targets (Fig. S5B). To validate this cooperation, we performed IF staining for RUNX2 and SREBP1 in TC cells. In both TPC1 and MDA-T41, SREBP1 resulted in its mature form with prevalent nuclear staining colocalizing with RUNX2 signal (Fig. 7A). Proximity Ligation Assay (PLA) confirmed that SREBP1 and RUNX2 are in proximity within the nuclei of TC cells (Fig. 7B; Fig. S5C-E). Co-IP experiments showed that RUNX2 binds to the mature (nuclear) but not to the precursor (cytoplasmic) isoform of SREBP1 both in TPC1 and MDA-T41 (Fig. 7C-D). Reverse Co-IP conducted on nuclear extracts reinforced these results (Fig. S5F), confirming the interaction between these two factors. To corroborate that this interaction is functional, we performed ChIP-seq (Fig. S5G) and RNA-seq analysis upon SREBP1 silencing (Fig. S5H) in TPC1 cells. ChIP-seq experiments identified 913 SREBP1 binding regions (Fig. S5G). By merging SREBP1 and RUNX2 ChIP-seq profiles, we identified 401 overlapping binding sites, representing 43.91% of the overall SREBP1-associated regions (Fig. 7E, Fig. S5I). Most of the RUNX2-SREBP1 overlapping regions were mapped within promoters, while only 31.90% within ENHs, preferentially exhibiting features of actively transcribed elements (25.10% of total) (Fig. 7F). These data confirmed our bioinformatic prediction (Fig. S5B), demonstrating that RUNX2 and SREBP1 co-occupy a subset of common regulatory regions. Figure 7G shows the co-localization of RUNX2 and SREBP1 on the promoter of FASN, selected as a representative target. We assigned the overlapping peaks to target genes and integrated these results with RNA-seq data from RUNX2 and SREBP1 KD cells, to filter for those that are transcriptionally affected by both TFs. We obtained a list of 99 target genes cooperatively regulated by both RUNX2 and SREBP1 (Fig. 7H). As expected, this list included a core of genes involved in lipid synthesis (Fig. S5J). We validated these results by using RNAi to silence both *SREBF1* and *RUNX2* (Fig. S5K) on MDA-T41 (Fig. 7I). Interestingly, we observed that RUNX2 expression was downregulated upon *SREBF1* KD (Fig. 7I), which is consistent with the previously identifiedSREs within two ENHs controlling *RUNX2* expression [34].

To confirm that RUNX2 is functionally required for the SREBP1 binding on common regulatory regions, we performed ChIP analysis for SREBP1 following RUNX2 KD (Fig. 7J). Notably, except for the promoter of *MSMO1*, RUNX2 silencing significantly decreased SREBP1 binding on these elements.

Our data demonstrated for the first time that RUNX2 controls *SREBF1* expression and functionally cooperates with this TF in transcriptional regulation, pointing to a central role of SREBP1 as mediator of the RUNX2 phenotype. To prove this hypothesis, we performed SREBP1 KD in TPC1 and tested the biological effect on cell growth, migration, and invasiveness. We observed that SREBP1 silencing impaired cell proliferation (Fig. 7K) and caused a strong reduction in cancer cell migration and invasion (Fig. 7L-M), recapitulating the RUNX2 KD phenotype and confirming its importance in TC aggressiveness. We also evaluated the biological effect of SCD, HMGCR, and FASN KD in TPC1 by functional assays (Fig. S5M-O). Silencing of each of the three enzymes resulted in coherent phenotypes that do not fully recapitulate the effect observed upon SREBP1 KD. This is coherent with a hierarchical organization of the RUNX2 transcriptional network, in which SREBP1 represents a key node in coordinating the module of genes involved in lipid anabolism. To validate this, we performed rescue experiments. Inducible overexpression of SREBP1a and SREBP1c – two major isoforms produced from the *SREBF1* gene via distinct TSSs and alternative splicing – in RUNX2-silenced cells restored the expression of downstream targets and rescued the migratory phenotype, confirming SREBP1 as a key mediator of RUNX2’s oncogenic functions (Fig. S6 A-F).

### SREBF1 expression correlates with increased aggressiveness of TC

By combining functional genomics with clinical data, we previously showed that the RUNX2 transcriptional program in TC is organized into 12 gene modules supervising specific biological functions, including metabolism regulation [27]. To weigh the contribution of these modules to clinical aggressiveness in vivo, we analyzed the expression of a panel of 239 genes, including 209 RUNX2-direct targets belonging to 11 out of 12 identified gene modules (Fig. S7A) in wo retrospective independent cohorts of TC and BC from our institution.

**Fig. 8:**
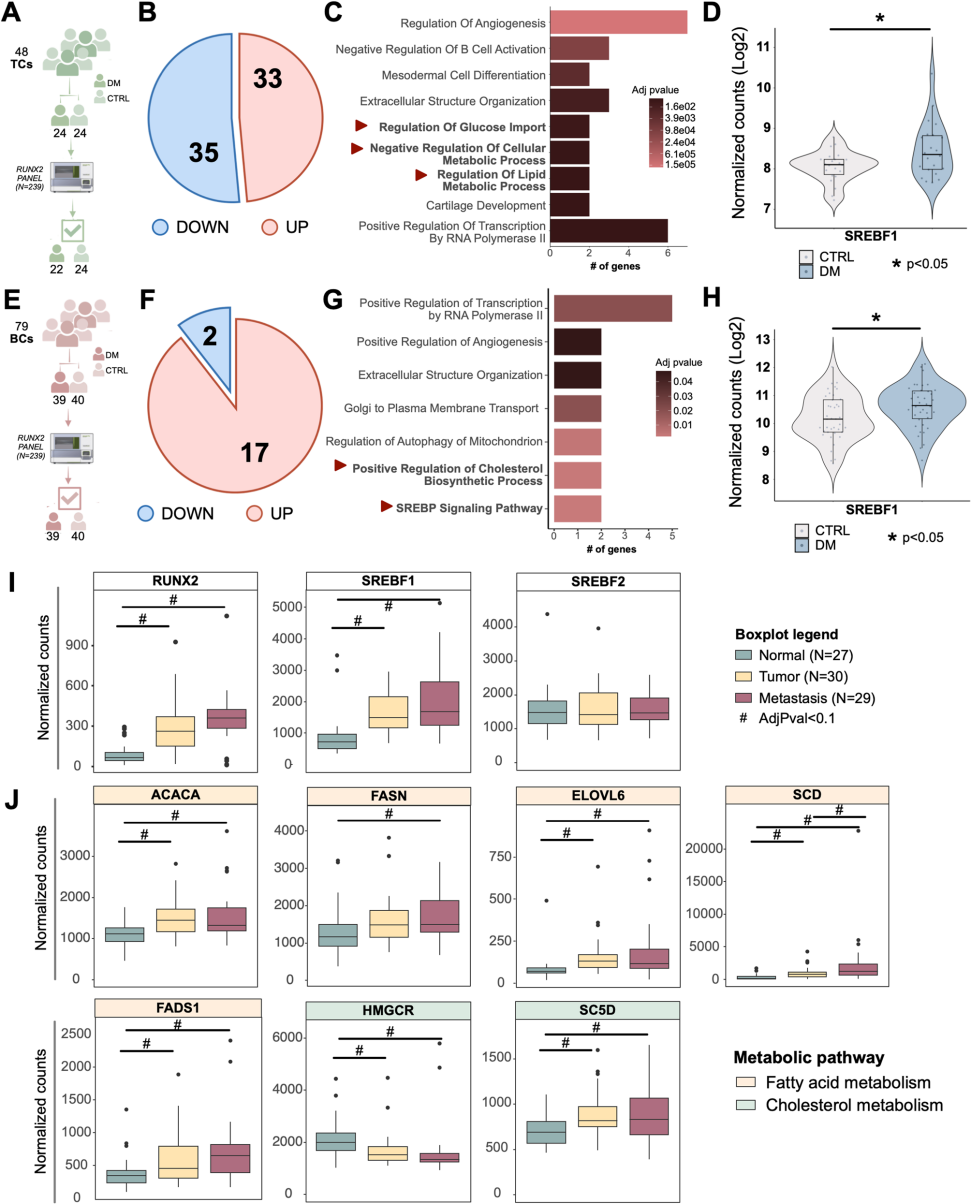
*SREBF1* expression is associated with increased metastatic potential and clinical aggressiveness in TC patients. (**A**) Workflow of Nanostring nCounter analysis on our internal TC cohort. (**B**) Pie charts showing the distribution of upregulated and downregulated genes in Distant Metastasis (DM) compared to control (CTRL) PTCs. (**C**) GO-BP enrichment analysis of upregulated genes. In bold are metabolism-related enriched terms. (**D**) Boxplot showing the normalized count of *SREBF1* in DM and CTRL PTCs. *p ≤ 0.05. (**E**) Workflow of Nanostring nCounter analysis on our internal BC cohort. (**F**) Pie charts showing the number of upregulated and downregulated genes in DM compared to CTRL TNBC. (**G**) GO-BP enrichment analysis of upregulated genes. (**H**) Boxplot showing the normalized count of *SREBF1* in DM and CTRL TNBCs. *p ≤ 0.05. I-J) Boxplots showing the normalized counts of *RUNX2*, *SREBF1*, *SREBF2* (**I**), and fatty acid and cholesterol (**J**) synthesis genes in the normal, tumor, and metastatic samples profiled by Shangy et al. [36]. The cohort includes 27 normal thyroid, 30 primary TC, and 29 TC lymph node (LN) metastasis

The TC cohort included *N* = 48 Papillary Thyroid Carcinomas (PTCs), of which 24 primary tumors that developed distant metastasis (DM) and 24 primary tumors that did not develop distant metastasis (CTRL) (Fig. 8A, Table S4). TC DMs are quite rare – 2–5% of the total cases [35] – making this cohort a significant set for the clinical validation of our data. By comparing DM vs. CTRL patients, we identified 68 differentially expressed genes (*p* ≤ 0.05), of which 35 were downregulated and 33 overexpressed in DM (Fig. 8B). Downregulated genes were associated with apoptosis and immunity (Fig. S7B), which we previously identified as RUNX2-gene modules correlated with reduced risk of aggressive disease [27]. Conversely, overexpressed genes were mainly enriched in GO categories linked to metabolism regulation (Fig. 8C), confirming the centrality of this function for TC clinical progression. *SREBF1* expression was significantly higher in DM samples as compared to CTRL (Fig. 8D), while *SREBF2* and *RUNX2* expression did not show significant differences (Fig. S7C). The BC cohort included *N* = 79 Triple Negative Breast Cancers (TNBCs), comprising *N* = 40 CTRL and *N* = 39 DM (Fig. 8E, Table S5). Differential expression analysis comparing DM vs. CTRL identified 19 altered genes, of which 17 upregulated and 2 downregulated (Fig. 8F). In line with the results obtained in TC, lipid synthesis-associated functions were significantly overrepresented in the DM-BC subset, confirming the association of this module with aggressive behavior (Fig. 8G). Indeed, the expression of *SREBF1* (Fig. 8H) - as well as the one of *SREBF2* (Fig. S7D) - was significantly upregulated in DM-BCs, reiterating the association of this TF with clinical aggressiveness.

We extended this evidence using a public RNA-seq dataset [36]. Compared to normal thyroid, the expression of both *RUNX2* and *SREBF1*, but not *SREBF2*, was significantly (adjusted p-value ≤ 0.1) increased in primary tumors and lymph-node metastases, with the latter showing slightly higher levels (Fig. 8I, Fig. S7E). Besides, key enzymes of fatty acids and cholesterol synthesis that we identified as RUNX2 targets (Fig. 2F-H) showed a similar trend (Fig. 8J, Fig. S7E). Correlation analysis between the expression of these genes and RUNX2 revealed significant positive correlations for *SREBF1*, *SREBF2*, *FASN*, and *SCD*, while *HMGCR* showed an opposite trend (Fig. S7F). To assess the generalizability of this correlation, we analyzed the correlation between *RUNX2* and a gene signature composed of the identified lipid-related targets (Fig. 2F) using TC, BC, and prostate cancer datasets from TCGA [37]. For all the tested tumor types, we found a positive correlation, supporting the validity of our data (Fig. S7G).

**Fig. 9:**
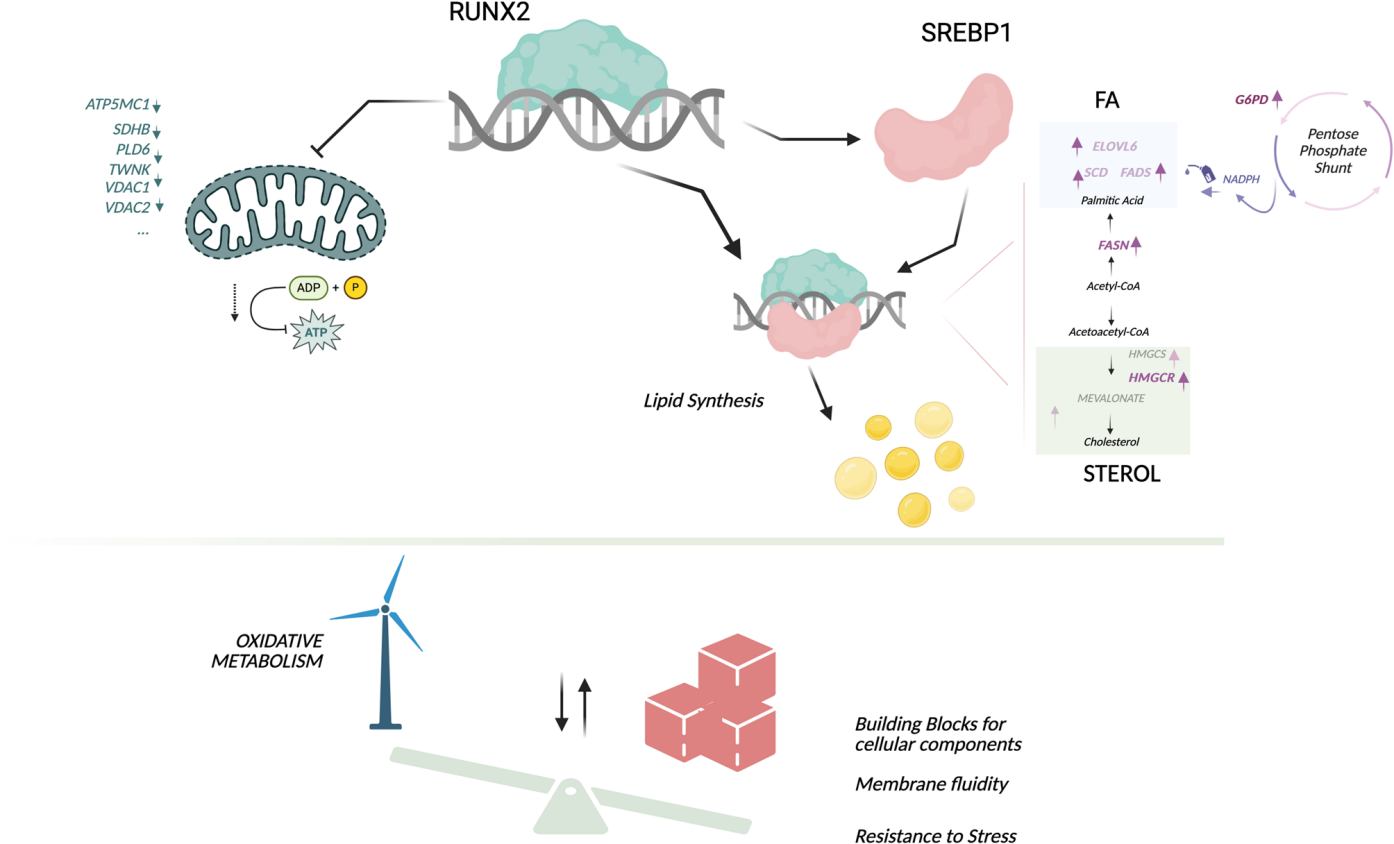
RUNX2 in the transcriptional control of cancer metabolic rewiring. Graphical representation of the molecular model proposed

In conclusion, our data provide strong evidence supporting the role of RUNX2 in the transcriptional control of cancer cell metabolism, and in particular in promoting lipid biosynthesis (Fig. 9). Using several approaches, we described for the first time the direct cooperation between RUNX2 and SREBP1, highlighting the importance of *de novo* lipid biosynthesis in the aggressiveness and clinical progression of TC.

## Discussion

Aberrant gene expression is a hallmark of cancer and sustains phenotypic plasticity [5–8]. Metabolic reprogramming is a part of this plasticity and represents an instrument for cancer cells to adapt to restrictive conditions and face environmental challenges [28, 33]. Like other embryonic TFs, RUNX2 is aberrantly reactivated in cancer, where it supports survival and metastasis [17]. In this work, we used a multi-omics approach to reconstruct the transcriptional landscape of RUNX2 in TC and link the downstream gene program to the biological processes that mediate its oncogenic function. We provided one of the first high-resolution annotations of the RUNX2-binding landscape in cancer, and we demonstrated a direct role of this TF in promoting cancer cell metabolic rewiring by altering the balance between oxidative respiration and anabolic pathways to support aggressiveness. On one side, we showed how the RUNX2 transcriptional activity directly affects mitochondrial structure and functionality by attenuating the expression of many mitochondrial proteins. Indeed, RUNX2 KD cells manifested a significant increase in respiratory capacity - in line with previous reports [38, 39] - and the appearance of highly fused mitochondria, a phenotype associated with increased energetic performance [40]. Interestingly, we previously reported that the Histone Deacetylase 6 (HDAC6) cooperates with RUNX2 in controlling gene expression in TC cells [41]. HDAC6 plays a crucial role in mitochondrial biology by regulating key processes like mitochondrial transport, fusion, and metabolism [42, 43]. It is tempting to speculate that the cooperation with HDAC6 could also be functionally relevant in RUNX2-mediated regulation of mitochondrial genes, but additional experiments will be required to fully address this possibility.

On the other side, RUNX2 directly promotes the expression of genes involved in lipid biosynthesis, together with those of the pentose phosphate shunt, which sustain cellular anabolism by producing NADPH [44]. In particular, we showed that key enzymes of fatty acid synthesis – including *ACACA*, *FASN*, and *SCD* – together with *HMGCR* – the rate-limiting enzyme of the mevalonate pathway of cholesterol synthesis- are direct targets of RUNX2 in both TPC1 and MDA-T41 cells. Notably, our ChIP-seq analyses showed RUNX2 enrichment on the regulatory regions of these genes in BC cell lines, further validating our genomics data generated in TC. Consistent with this, untargeted lipidomics analysis revealed a profound alteration of the lipidic profile following RUNX2 silencing in both TC and BC cell models. Accordingly, untargeted metabolomics performed in the same conditions identified decreased intracellular concentration of mevalonate and alterations in lipid molecules. Lipid metabolism and mitochondrial function are closely linked. Mitochondria play a crucial role in lipid metabolism [45–47], both catabolizing lipids for energy and providing Acetyl-CoA for fatty acid and cholesterol synthesis [45]. Our data suggest that through its transcriptional activity -both as activator and repressor of gene expression- RUNX2 can synchronize these functions to ensure the most advantageous metabolic asset for tumor cells.

D*e novo* lipogenesis is an essential process that is drastically altered in cancer cells. Lipid molecules represent basic components for cell membranes and efficient energy sources. Furthermore, mounting evidence shows how lipids work as signaling molecules in several oncogenic pathways, shaping numerous features of cancer cells and the surrounding microenvironment [48–51]. Cells have evolved an elaborate machinery to sense lipid availability and activate *de novo* lipogenesis to keep up with cellular needs. This sensing mechanism culminates with the cleavage of SREBP1 and its translocation to the nucleus, where it drives the expression of enzymes involved in lipid biosynthesis [52, 53]. Here, we show for the first time that *SREBF1* is a direct target of RUNX2 and that they functionally cooperate to regulate the expression of a gene module that executes lipid synthesis functions. This type of organization fits well within the hierarchical model of the RUNX2 transcriptional landscape that we recently proposed, according to which RUNX2 coordinates distinct biological functions by regulating and cooperating with bottleneck TFs [27]. Perturbation of these intermediate regulators propagates to the downstream gene module, affecting the associated biological function. Coherently, we observed that silencing of individual enzymes involved in lipid metabolism—specifically FASN, SCD, and HMGCR—is not sufficient to fully replicate the phenotype of RUNX2 KD, while the knockdown of SREBP1, their upstream regulator, successfully phenocopied the RUNX2 silencing in TC cells, strongly impacting on cancer cell aggressive behaviors.

Several groups reported the upregulation of *SREBF1* in cancer, underlying its pro-tumorigenic roles [54–56]. In line with this, our analyses showed that *SREBF1* is upregulated in TCs and BCs that developed metastasis, both in an internal case-control cohort and in publicly available datasets. As structural components of the plasma membrane, lipids are responsible for contributing to membrane tension, rigidity, and shape. During metastatic spreading, the cell membrane behaves as a dynamic and highly plastic structure that provides resistance to mechanical tensions and ductility to allow cell motility. This plasticity is achieved through the fine modulation of the lipid species that compose the membrane [57–59]. Therefore, the cooperation with SREBP1 in regulating lipogenesis genes raises the intriguing possibility that RUNX2 could influence cell membrane composition, adding a new dimension to the multifunctional framework of its metastatic functions. Further studies are needed to understand how the transcriptional action of RUNX2 affects cell membrane plasticity and the extent to which it contributes to the process of TC metastasis in vivo.

In conclusion, our multi-omics analysis generated extensive data that highlights a previously uncharacterized role for the transcription factor RUNX2 in coordinating the expression of genes affecting mitochondrial function and lipid homeostasis. Our data indicate that RUNX2 – through its widespread transcriptional activity – is a key factor for a balanced gene expression that fosters cancer cell fitness through the downregulation of mitochondrial respiration and the promotion of anabolic pathways like *de novo* lipogenesis. We showed that this function constitutes a distinctive feature of the RUNX2 transcriptional program in thyroid and breast cancers, and we suppose it may represent an additional level of phenotypic plasticity through which RUNX2 promotes cancer metastasis.

## Materials and methods

### Cell cultures

TPC1 cells, kind gift of Prof. Massimo Santoro (University of Naples, Naples, Italy), MDA-MB231, obtained from Dr. Adriana Albini, and HEK293T, purchased from American Type Culture Collection (ATCC, Manassas, VA, USA), were cultured in DMEM-Glutamax (Gibco, Thermo Fisher Scientific, Waltham, MA, USA) with 10% Fetal Bovine Serum (FBS) and 1% penicillin-streptomycin (P/S) (Gibco, Thermo Fisher Scientific Waltham, MA, USA). MDA-T41 and Hs578T cells were purchased from ATCC and grown in RPMI-1640 (EuroClone S.p.A., Milan, Italy) with 1% non-essential amino acids (Gibco, Thermo Fisher Scientific Waltham, MA, USA), 10% FBS, 1% P/S, and DMEM-Glutamax with 0.01 mg/ml human insulin (Sigma-Aldrich, St. Louis, Missouri, USA), 10% FBS, and 1% penicillin-streptomycin, respectively. All cell lines were grown at 37 °C/5% CO2 and routinely tested for mycoplasma infection. Cells were authenticated by SNP profiling at Multiplexion GmbH (Heidelberg, Germany) in January 2023.

### Plasmids and cell lines establishing

CRISPR-interference (CRISPRi) was performed as previously described (27). SgRNAs against targets of interest, and a non-targeting (NT) sgRNA (control) were cloned in Plv-hU6-sgRNA hUbC-dCas9-KRAB-T2a-Puro (AddGene #71236, gift from Charles Gersbach [60] (Addgene, Watertown, MA, USA) with Esp3I (Thermo Fisher Scientific).

Inducible RUNX2-silencing was obtained by RNA interference. RUNX2-targeting shRNA was cloned into Tet-PLKO-puro (Addgene #21915, gift from Dmitri Wiederschain [61] and Tet-PLKO-EGFP [62]. Sequences of sgRNAs and shRNA are listed in Table S6.

Plasmids for inducible SREBP1 overexpression were obtained by subcloning SREBP1a and SREBP1c sequences from pcDNA3.1-2xFLAG-SREBP-1a (Addgene #26801, gift from Timothy Osborne [63] and pcDNA3.1-2xFLAG-SREBP-1c (Addgene #26802, gift from Timothy Osborne), respectively, into PCW Empty Vector (EV) (Addgene #184708). Inserts were removed from pcDNA3.1 plasmids by digesting with NheI and MssI (PmeI). PCW was digested with NheI and KspaI (HpaI) for directional cloning. All the restriction enzymes were purchased from Thermo Fisher scientific.

Lentiviral particles were produced in HEK293T co-transfected with transfer vectors, pRSV-Rev, pMDLg/pRRE, and pMD2.G (AddGene #12253, #12251, and #12259, gift from Didier Trono) using Lipofectamine 2000. Infected cells were selected with 1 µg/ml puromycin (Merck Millipore, Burlington, MA, USA) for 5 days or sorted for their GFP brightness using FACS Melody (BD Bioscences). For inducible systems, cells were treated with 100 ng/ml doxycycline for 48 h to induce gene expression or silencing.

### RNA-extraction and RNA-sequencing (RNA-seq)

Total RNA was extracted with Maxwell RSC Simply RNA Cells (Promega, Madison, Wisconsin, USA) or RNeasy Plus Mini Kit (QIAGEN, Germantown, MD, USA).

RNA-sequencing libraries were obtained starting from 100 ng of total RNA following Illumina Stranded TotalRNA PrepLigation with Ribo-zero Plus protocol (Illumina, San Diego, California, USA). Sequencing was performed using Illumina NextSeq high-output cartridge (paired-stranded, read length 75 bp).

### qRT-PCR

RNAs were retrotranscribed with iScript cDNA kit (Biorad, Hercules, California, USA). Quantitative Real-Time PCR (qRT-PCR) was performed using Sso Fast EvaGreen Super Mix (Biorad, Hercules, California, USA) in the CFX96 Real-Time PCR Detection System (Biorad, Hercules, California, USA). Relative expression was calculated using the 2- ΔCt method by normalizing to the reference genes Actin B (ACTB), and Cyclophilin A (PPIA). Sequences of qPCR primers are listed in Table S7 and S8. Paired two-tailed T-Test was performed with GraphPad Prism 9 to assess statistical significance.

### Western blot

Total proteins were extracted using Passive Lysis Buffer (Promega, Madison, Wisconsin, USA) supplemented with Protease Inhibitor (PI) cocktail (Roche, Basel, Switzerland). 20–30 µg of proteins were loaded on Mini-Protean TGX pre-cast gels (Biorad, Hercules, California, USA) and SDS-PAGE was performed using the Biorad apparatus (Biorad, Hercules, California, USA). Immunoblot detection was performed with the appropriate HRP-conjugated secondary antibodies (GE Healthcare, Piscataway, NJ, USA) and Clarity Western ECL substrate (Bio-Rad, Hercules, CA, USA). Antibodies used for western blot analysis are listed in Table S9.

### Chromatin immunoprecipitation (ChIP)

ChIP-seq was performed as previously described [27]. Libraries were obtained following the ThruPLEX DNA- Seq Kit (Takara Bio Ink, Kusatsu, Japan). For each experiment, three independent replicates were sequenced on Illumina NextSeq500 high-output cartridge (single-end, read length 75 bp).

ChIP-qPCR on RUNX2 KD and CTRL cells was performed with SimpleChIP^®^ Enzymatic Chromatin IP Kit with Magnetic Beads (Cell Signaling Technology, Danvers, MA, USA) as previously described [64]. Immunoprecipitation was performed with 0.5 µg of anti-SREBP-1 (E9F4O, Cell Signaling Technology) or normal rabbit IgG (2729, Cell Signaling Technology). SREBP1 enrichment on selected regions was evaluated by qRT-PCR. Each qRT-PCR value was normalized over the appropriate input control. Relative fold enrichment of RUNX2 KD over control cells was calculated for each target and represented on the graph. A paired two-tailed T-Test was performed with GraphPad Prism 9 to assess statistical significance.

### Untargeted metabolomics

Untargeted metabolomics was performed by the Laboratory of Mass Spectometry (Head Dr. Roberta Pastorelli), Department of Environmental Health Sciences, Istituto di Ricerche Farmacologiche Mario Negri IRCCS (Milan, Italy). Metabolites from TPC1 and MDA-T41 cells were quenched and extracted as previously described [65]. Flow injection analysis high-resolution mass spectrometry (FIA-HRMS) was used for untargeted metabolomics of TPC1 and MDA-T41 cells as previously described [66]. All data were processed and analyzed in Matlab R2016a (The Mathworks, Natick, MA, USA) using an in-house developed script [67]. The Wilcoxon-Mann-Whitney test was used to identify statistically significant altered metabolites.

### Immunofluorescence staining

Immunofluorescences (IFs) were performed on cells plated on 12 mm diameter coverslips.

*TOM20 IF*. Cells were fixed with 1% glutaraldehyde (CARLO ERBA Reagents S.r.l., Milan, Italy) for 10 min RT and then incubated in 1 mg/ml NaBH4 (Sigma Aldrich, St. Louis, Missouri, USA) for 15 min. Permeabilization was performed with 0,25% Triton X-100 for 10 min. Cells were blocked with 10% FBS for 30 min and then incubated with TOM20 antibody, 1 h 30 min RT (Table S9). Alexa fluor 488 anti-rabbit secondary antibody (#A-11008, Thermo Fisher Scientific, Waltham, MA, USA) diluted 1:500 was mixed with 1:500 Alexa-Fluor 647 Phalloidin (Thermo Fisher Scientific, Waltham, MA, USA) and incubated 1 h RT. After DAPI-staining for 5 min RT, coverslips were mounted using ProLong Gold antifade mounting media (Thermo Fisher Scientific, Waltham, MA, USA).

*RUNX2/SREBP1 IF*. Cells were fixed with 4% paraformaldehyde (PFA) for 15 min at RT. Permeabilization was performed with 0.1% Triton X-100 for 2 min. Cells were blocked with 20% FBS in PBS 2% BSA for 1 h. Primary antibodies were diluted in PBS 2% BSA and incubated 2 h RT. Alexa Fluor 488 goat anti-mouse IgG (#A11001, Thermo Fisher Scientific, Waltham, MA, USA) and Alexa Fluor 594 anti-rabbit (#A11012, Thermo Fisher Scientific, Waltham, MA, USA) secondary antibodies, both diluted 1:1000 in PBS 2% BSA, were incubated 1 h RT. Nuclei were stained with DAPI.

Images were acquired with Nikon Eclipse (Nikon, Tokyo, Japan) or EVOS M5000 Imaging System (Thermo Fisher Scientific, Waltham, MA, USA). Mitochondria morphology was analyzed with the Fiji-plugin Mitochondria Analyzer. Unpaired two-tailed T-Test was performed with GraphPad Prism 9 to assess statical significance.

### Proximity ligation assay (PLA)

Duolink In Situ Red Mouse/Rabbit PLA kit (# DUO92101, Sigma Aldrich, St. Louis, Missouri, USA) was used following the manufacturer’s instructions. Fixed cells were incubated overnight with 1:200 anti-SREBP1 (Rabbit, #14088-1-AP, Proteintech) and 1:100 anti-RUNX2 (Mouse, sc-390351, Santa Cruz Biotechnology). PLA probes were incubated for 1 h at 37 °C. Ligation was performed for 30 min at 37 °C. Alexa Fluor 488 Phalloidin (Thermo Fisher Scientific, Waltham, MA, USA) was used to stain actin filaments and DAPI to stain nuclei.

### Resipher analysis

Oxygen Consumption Rate (OCR) was monitored using the RESIPHER system (Lucid Lab, Rogers, Minnesota, USA) for 72 h. OCR values were normalized on cell number. The OCR of RUNX2 KD relative to control was calculated and represented in the graphs. Unpaired two-tailed T-Test was performed with GraphPad Prism 9 to assess statistical significance.

### Seahorse analysis

Mitochondrial functionality was analyzed with the Seahorse Mitostress Test (Agilent Technologies, Santa Clara, California, USA). The assay was run on the Agilent XF24 or XF96 analyzer (Agilent Technologies, Santa Clara, California, USA). 50,000 TPC1 and 60,000 MDA-MB231 cells were plated on Seahorse XF24 microplates. 10,000 MDA-T41 cells were plated on Seahorse XF96 culture microplates. Cells were treated with 1.5 µM Oligomycin, 2.5 µM FCCP, and a mix of 5 µM Rotenone and 2,5 µM Antimycin A. OCR values were normalized on cell number. Basal OCR was calculated as a difference between the OCR value before Oligomycin administration and the OCR value after Rotenone + Antimycin injection (non-mitochondrial OCR). Maximum respiratory capacity was calculated by subtracting the non-mitochondrial OCR from the OCR value after FCCP treatment. OCR linked to ATP production was calculated as the difference between OCR values before and after oligomycin administration. OCR folds of RUNX2 KD over control cells were calculated and represented on histograms. Unpaired two-tailed T-Test was performed with GraphPad Prism 9.

### Untargeted lipidomics

Lipidomics was performed by the Wistar Institute Proteomics and Metabolomics Shared Resource as described previously [68]. Samples were analyzed by LC-MS/MS on a Thermo Scientific Q Exactive HF-X mass spectrometer coupled to a Vanquish UHPLC system. Lipidomics data were processed using LipidSearch 4.2 (Thermo Scientific). Statistical analysis was performed using Perseus 2.0.9.0 [69]. Data were log2-transformed, and pairwise comparison was performed for RUNX2 KD vs. control using Student’s t test with Benjamini-Hochberg correction for multiple hypothesis testing. For lipid classes, normalized MS peak areas were summed for all lipid species in a given class in each sample, and pairwise comparison was performed using the Student t-test.

### Co-Immunoprecipitation (Co-IP)

Co-IP of endogenous RUNX2 and SREBP1 was performed starting from 40 to 60 × 10^6^ cells.

*RUNX2-IP.* Proteins were extracted with a modified RIPA Buffer (50mM Tris-HCl pH 7.5, 1mM EDTA, 150mM NaCl, 0.5% NP-40, 5% Glycerol, 1 mM DTT, protease and phosphatase inhibitors) incubated at 4 °C, 30 min on a rotating wheel. Lysate was clarified by centrifugation (13000 rpm, 10 min, 4 °C) and pre-cleared with 20 µl Dynabeads Protein G magnetic beads (Thermo Fisher Scientific, Waltham, MA, USA). Precleared lysate was quantified with Bradford (Biorad, Hercules, California, USA), and 4–6 mg of protein was incubated overnight with 5 µg anti-RUNX2 (Rabbit, DI7LF, Cell Signaling Technologies) or Normal rabbit IgG (Rabbit, 2729, Cell Signaling Technologies). Immunocomplexes were precipitated with 20 µl Dynabeads Protein G magnetic beads for 2 h at 4 °C while rotating. After three washes with TBS (50 mM Tris-HCl pH 7.5, 150 mM NaCl), the beads were resuspended in 20 µl of Laemmli Buffer without β-mercaptoethanol, incubated for 10 min at 95 °C while shaking.

*SREBP1-IP*. The reverse Co-IP was performed on cross-linked nuclei. For cell fractionation, cells were lysed with Nuclear Isolation Buffer (256 mM sucrose; 8 mM Tris-HCl pH 7.5; 4 mM MgCl_2_; 0.8% Triton X-100, 0.2X PBS) supplemented with protease and phosphatase inhibitors and incubated on ice for 20 min. Nuclei were isolated by centrifuging at 3000 rpm for 15 min. After two washes with Buffer B (10 mM Hepes pH 7.9; 1.5 mM MgCl_2_; 10 mM KCl, protease and phosphatase inhibitors), nuclei were crosslinked with 1% formaldehyde for 10 min, followed by quenching with 0.125 M glycine. Crosslinked nuclei were lysed with Nuclei Lysis Buffer (50 mM Tris-HCl pH 8.0, 10 mM EDTA, 1% SDS) supplemented with protease inhibitors and subsequently sonicated using a Bioruptor^®^ Pico sonicator (Diagenode, Denville, NJ, USA). Lysate was clarified by centrifugation at 14,000 rpm for 10 min at 4 °C and then diluted 1:10 with Dilution buffer (16.7 mM TRIS HCl pH8; 1.1% TRITON; 0.01% SDS; 1.2 mM EDTA; 167 mM NaCl) supplemented with protease inhibitors. Immunoprecipitation was performed as described for RUNX2-IP, using 5 µg of anti-SREBP-1 antibody (rabbit polyclonal Ab, 14088-1-AP, Proteintech) or normal rabbit IgG (Rabbit, 2729, Cell Signaling Technology).

Each IP and 70–80 µg of input were loaded onto Mini-Protean TGX pre-cast gels (Biorad, Hercules, California, USA) for SDS page. Immunoblot detection was performed with anti-RUNX2 (Mouse, sc-390351, Santa Cruz Biotechnology) and anti-SREBP1 (Mouse, MA5-16124, Thermo Fisher Scientific) antibodies (Table S9). For fractionation controls, the following antibodies were used: anti-α-Tubulin (Mouse, sc-8035, Santa Cruz Biotechnology), anti-Phospho-Rpb1 CTD (Ser5) (Rabbit, mAb, 13523, Cell Signaling Technologies).

### SiRNA transfection

MDA-T41 were transfected with 20 nM of validated silencer select SREBF1 (#4392420, Thermo Fisher Scientific Waltham, MA, USA) or negative control (#4390847, Thermo Fisher Scientific Waltham, MA, USA) siRNA. siRNAs were delivered by reverse transfection using RNAiMax Lipofectamine reagent (Thermo Scientific, Waltham, MA, USA).

### Proliferation assays

1,000 cells/well were seeded in a 96-well plate and labeled with Incucyte^®^ Nuclight Rapid Red Dye (1:1000, Sartorius AG, Göttingen, Germany). Nuclei number was evaluated every 12 h for 4 days using the Incucyte^®^ Live-Cell Analysis System *(Model S3; Sartorius AG*,* Goettingen*,* Germany).*

### Wound healing and invasion assays

25,000 cells/well were seeded in an IncuCyte^®^ ImageLock 96-well plate (Sartorius AG, Göttingen, Germany). The following day, cells were treated with 0.01 mg/mL mitomycin C (M4287, Sigma-Aldrich, St. Louis, Missouri, USA) for 2 h. Scratch wounds were made with the Incucyte^®^ WoundMaker Tool (Sartorius AG, Göttingen, Germany).

For *wound healing*, cell migration was assessed by evaluating scratch Wound Confluence every 3 h for 2 days.

For *invasion assays*, after scratching, cells were covered with a 1:5 dilution of Matrigel (Corning, Corning, NY, USA). Cell invasion was assessed by analyzing Relative Wound Density every 3 h for 5 days.

All analyses were performed with the Incucyte^®^ Live-Cell Analysis System (Model S3; Sartorius AG, Göttingen, Germany).

### Patients’ selection and Nanostring analysis

Two independent retrospective case-control cohorts of 48 PTCs and 79 TNBC were selected from the archive of the Pathology Unit of our Institution. The cohorts were constructed as case:control, in which cases were primary tumors from patients that developed distant metastasis (DM) while controls (CTRL) were selected as patients that did not develop distant metastasis in follow-up of at least 10 years [70]. The TC cohorts included *N* = 24 DM and *N* = 24 CTRL PTCs, whose clinical data are shown in Table S4. The BC cohorts included *N* = 39 DM and *N* = 40 CTRL TNBC, whose clinical features are reported in Table S5. Slides of 5 μm FFPE tissue from histological resections of the primary lesion were retrieved and total RNA extracted by Maxwell RSC RNA FFPE kit (Promega, Madison, Wisconsin, USA). The expression of a custom panel of 239 genes including *RUNX2*, 209 RUNX2 target genes, and 29 housekeeping genes was analyzed using the Nanostring nCounter (Nanostring Technologies, Seattle, Washington, USA). Gene counts were performed using nSolver Analysis Software 4.0 (Nanostring Technologies, Seattle, Washington, USA) as previously described [71].

### Bioinformatic analysis

Sequencing data quality was assessed using the FastQC v0.11.8 software (www.bioinformatics.babraham.ac.uk/projects/fastqc/). A detailed description of bioinformatic methods employed is available in the Supplementary Information.

### Statistical analysis

Statistical analysis was performed using GraphPad Prism Software (version 9.3.0 for Windows, GraphPad Software, San Diego, CA, USA). Statistical significance was determined using the T-test (two-tailed, paired or unpaired). Each experiment was replicated at least three times. For NGS experiments, statistical analyses were performed on R version 4.1.0 unless otherwise specified.

## Supplementary Information

Below is the link to the electronic supplementary material.

Supplementary Material 1

## Data Availability

NGS sequencing data generated and analyzed during the current study are available in the ArrayExpress repository.RNAseq experiments: E-MTAB-11049, E-MTAB-13777.ChIP-seq: E-MTAB-11051, E-MTAB-11052, E-MTAB-11050, E-MTAB-14152.
