# Supplementary material for: RUNX2 cooperates with SREBP1 to rewire cancer metabolism and promote aggressiveness
Source: J Exp Clin Cancer Res. 2025 Oct 31;44:298. doi: 10.1186/s13046-025-03549-7 (PMC12577400; doi:10.1186/s13046-025-03549-7)
Supplement: Supplementary file 1 — Supplementary Material 1 [file 13046_2025_3549_MOESM1_ESM.pdf]

Supplementary Material and Methods

Bioinformatic Analysis

RNA-seq. High-quality pair-end reads were aligned to the human reference transcriptome (GRCh38, Gencode release 30) using STAR v2.7. Transcript abundance was estimated with the RSEM algorithm (1.3.1). Differential analysis was performed with the R (3.6.0) package DESeq2 (1.25.14) on raw counts using default parameters. Genes with low reads (less than 10 counts across all samples) were excluded and a False Discovery Rate (FDR) of 0.1 was adopted as a significance threshold.

ChIP-seq. Residual adapter sequences were removed using Trimmomatic (0.39) software. Filtered reads were aligned to the human reference genome (GRCh38/hg38 assembly) by using Bowtie2 (2.3.5.1). Duplicated and unmapped reads were removed with Picard tool (http://broadinstitute.github.io/picard) and Samtools (1.9) (http://samtools.sourceforge.net/). To find regions of ChIP-seq enrichment over Input, peak calling was performed using MACS2 (2.1.3.3) with default parameters and considering a cut-off of $q \leq 0.05$. Consensus peakset was obtained considering only peaks that overlapped in at least 2 out of 3 replicates. Peak-to-target assignment was performed on R (3.6.0) using the ChIPseeker (1.21.1) package to assign peaks to the nearest genes, according to TxDb.Hsapiens.UCSC.hg38.knownGene annotation (3.4.6), with default parameters (TSS window of ±3 kb).

Functional enrichment analyses were performed by enrichR R package using Gene Ontology - Biological Process (GO-BP) and Reactome as reference databases.

Terms with adjusted p-value≤0.05 (adjusted according to the Benjamini-Hochberg correction) were considered significantly enriched.

Chromatin states. Chromatin states for both cell lines (TPC1 and MDA-T41) were identified by ChromHMM (38)(v1.23) considering the aligned reads from the three histone marks (H3K4me1, H3K4me3, and H3K27ac) and RNA-PolII, using the default parameters. Non-overlapping 200-bp bins were used for model learning and segmentation. An 11-state model was chosen for downstream analysis. The labels were assigned to each state after the inspection of the emission matrix. To functionally characterize the RUNX2-associated regions, we i) considered the overlap of each peak with all possible chormHMM states; ii) summed up these overlaps by chromHMM state (i.e., the coverage), and iii) defined the percentages of coverage by dividing them by the sum of all RUNX peaks. Notably, almost all RUNX2-peaks overlap with only one chromHMM state major group (i.e., promoter, enhancer, transcription, or undetermined). The genome-wide coverage percentages were obtained by considering the total coverage of each chromHMM state divided by the genome length.

SRE motif search. Motif Search analysis was performed by applying FIMO (5.0.2) algorithm. RUNX2 peaks associated with selected lipid metabolism genes and assigned to promoter or enhancer ChromHMM states were selected. A region of 500bp around peak center was considered for FIMO input. SREBF1 motifs were retrieved from JASPAR CORE non-redundant vertebrates and HOCOMOCO CORE v12 datasets.

SREBP1 and RUNX2 cooperation. Under the null hypothesis of independence (*i.e.* absence of cooperation between RUNX2 and SREBF1 in a gene region), the shrunken log2 FCs of the differential expression analysis (KD vs. control, using a normal priori, DESeq2 package) are distributed as a bivariate normal with zero

covariance. All the genes for which the shrunken log2 FCs resided outside the 95% confidence region, based on the parameters (means and variances) estimated on non-target genes, were considered as cooperatively regulated by both TFs (rejection of null hypothesis).

Public RNA-seq data. The expression matrix of raw counts of the cohort profiled by Sanghi A. et al (Sanghi, A. et al, *Nat Comm*, 2021) was downloaded from GEO (accession number GSE162515). Differential analysis was performed by DESeq2 (1.32.0) R package on the downloaded raw counts using default parameters. A False Discovery Rate (FDR) of 0.1 was considered to select significantly deregulated genes. Gene expression matrix normalized by size factors was retrieved for boxplots in Figure 7.

Correlation Analysis. Correlation analyses were conducted using the Pearson method. For in-house data analysis, the R Corrplot package was employed. Correlation between RUNX2 (ENST00000359524.7) and a signature of its lipid-metabolism target genes (as listed in Fig. 2F) was assessed using the online platform GEPIA2 (doi: 10.1093/nar/gkz430).

Supplementary Figures

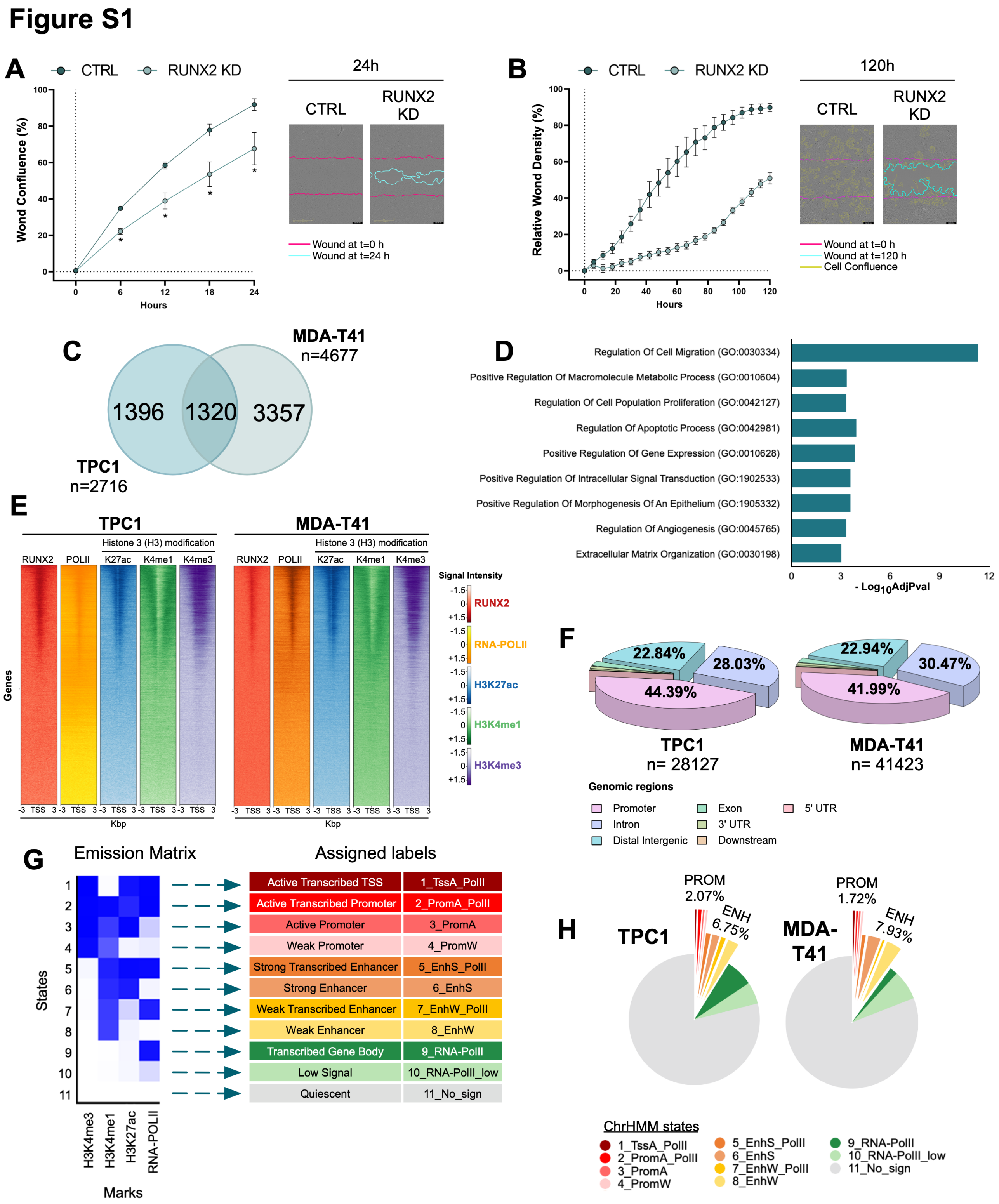

Figure S1. A) Wound healing assay to assess migration in CTRL and RUNX2 KD MDA-T41 cells. The graph represents the wound confluence Mean±SEM of five independent replicates. On the right are representative images showing the wound area at the experiment endpoint (24 h). Scale bar 600 µm. *p≤0.05. B) Invasion assay in CTRL and RUNX2 KD MDA-T41. Data are presented as Mean ± SEM from a technical triplicate of

a representative experiment. Representative images on the right show the wound area and cell confluence at the experiment endpoint (120 hours). Scale bar 600 µm. C) Venn diagram highlighting the number of common RUNX2 KD DEGs in TPC1 and MDA-T41. D) GO-BP enrichment analysis of TPC1/MDA-T41 common DEGs. E) ChIP-signal around TSS of RUNX2, RNA-PolII, H3K27ac, H3K4me1, and H3K4me3 in TPC1 (left) and MDA-T41 (right). F) Pie charts showing the RUNX2-peaks assignment to genomic regions in TPC1 (left) and MDA-T41 (right). The percentage of the most enriched genomic regions is shown in the graph. G) Emission probabilities matrix and schematic representation of Chromatin States definition by ChromHmm. 11 states were defined based on the different enrichment levels of the analyzed chromatin markers. H) Pie chart showing the genome-wide distribution of the 11 chromatin states in TPC1 (left) and MDA-T41 (right).

Figure S2

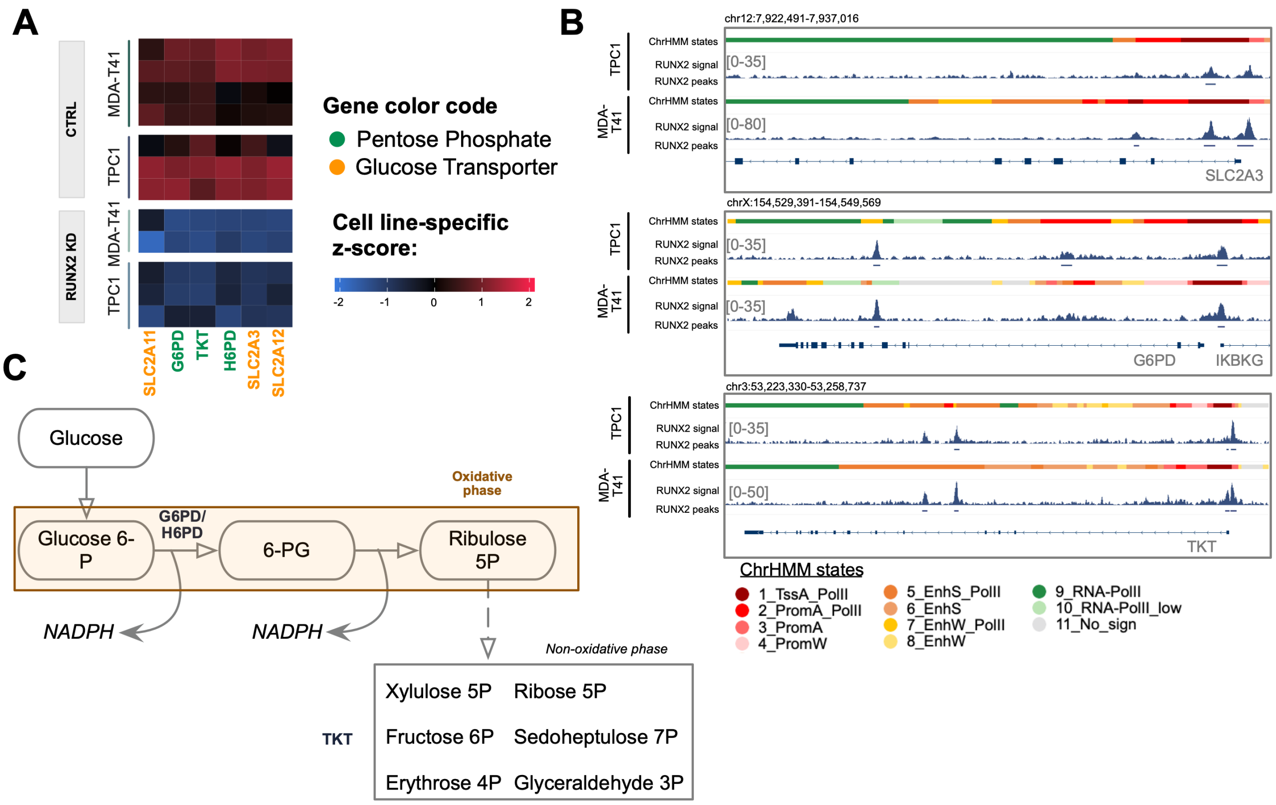

Figure S2. A) Gene expression heatmap of RUNX2-direct target genes encoding for glucose transporters (yellow) and pentose-phosphate enzymes (green). B) Representative IGV tracks showing the RUNX2 binding on the promoter of *SLC2A3*, *G6PD*, and *TKT*. C) Graphical representation of pentose-phosphate pathway, with representative enzyme identified as RUNX2-direct targets (TPC1 dataset).

Figure S3

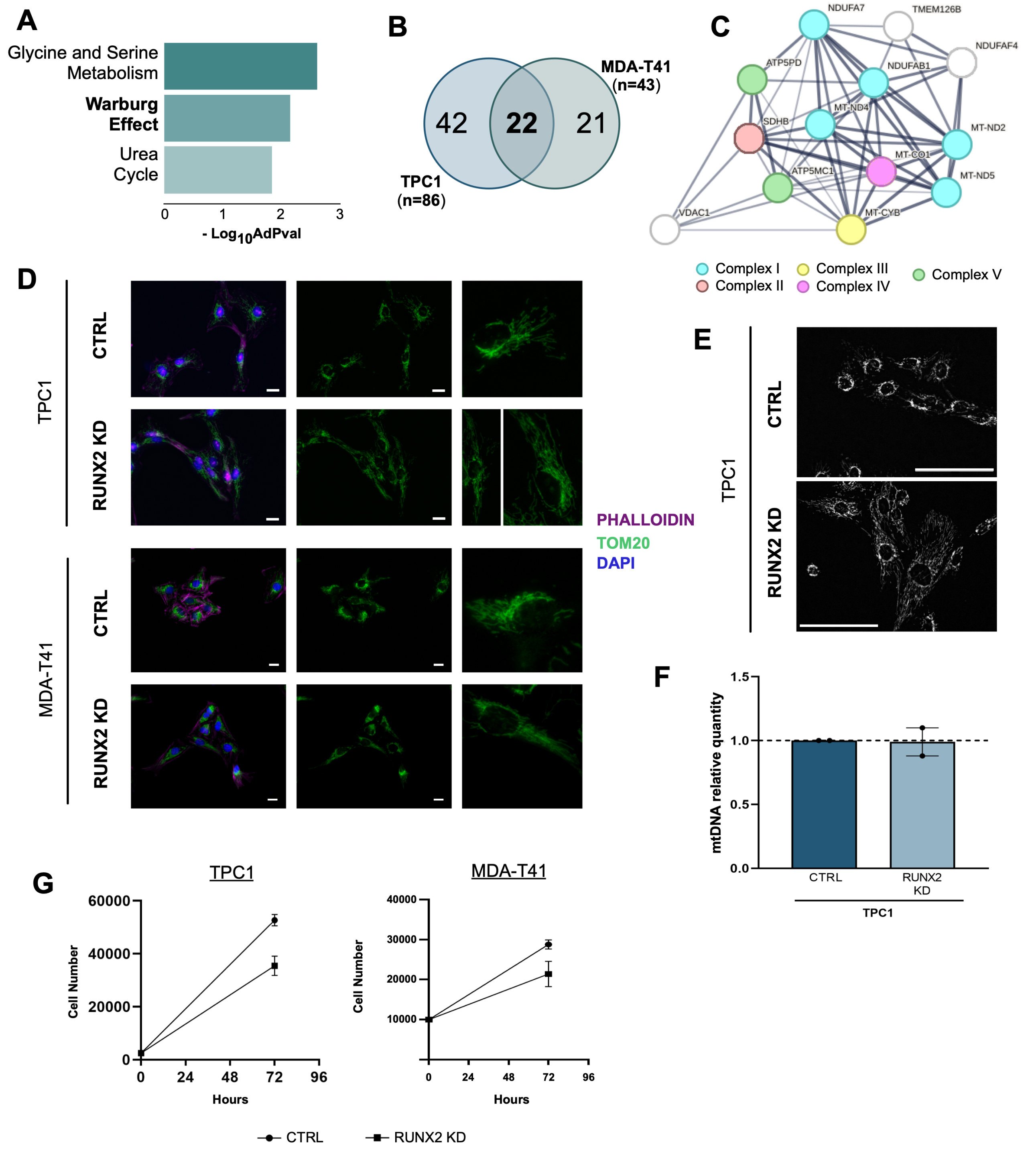

Figure S3. A) MetaboAnalyst pathway enrichment analysis of altered metabolites upon the RUNX2 loss in MDA-T41. B) Venn diagram highlighting the number of common metabolites altered upon the RUNX2 KD in TPC1 and MDA-T41. C) STRING analysis of representative upregulated genes encoding for mitochondrial complex subunits in RUNX2 KD TPC1. D) Representative images of TOM20 (green) and phalloidin (magenta) staining on RUNX2 KD and CTRL TC cells. Scale Bar=20 µm. E) Live imaging analysis of TMRM-stained mitochondria in RUNX2 KD and CTRL TPC1. Scale Bar=50 µm. F) mtDNA relative

quantification by Real-Time PCR. Histograms show the Mean±SEM of 2 independent experiments in TPC1. G) Resipher analysis. Estimation of cell number after 72 h of cell growth. Graphs show the cell number relative to the representative experiments shown in Figure 4 E, G.

Figure S4

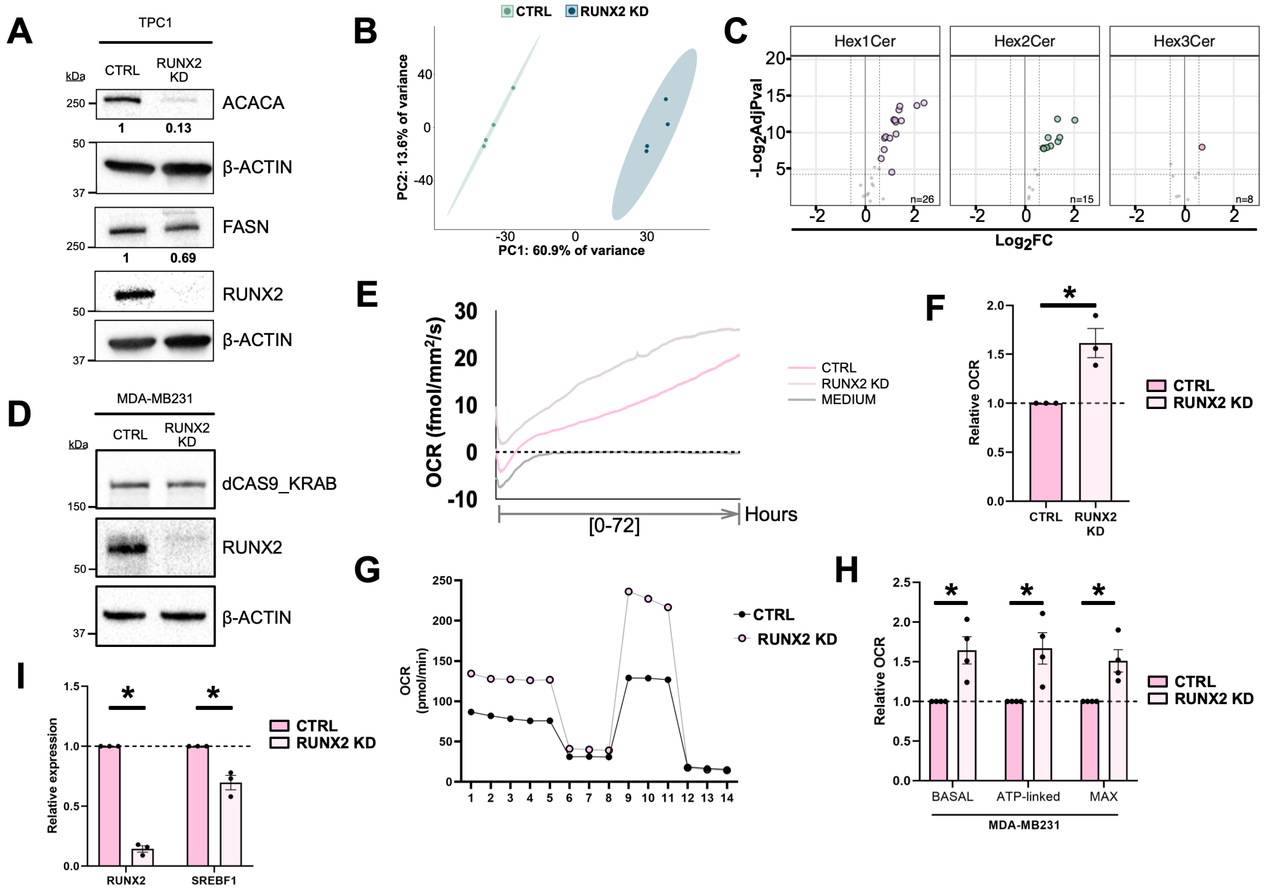

Figure S4. A) Western Blot showing ACACA and FASN decrease upon RUNX2 silencing. Quantification normalized on β-Actin was performed using ImageJ, and the fold over CTRL was indicated in the figure. B) Principal Component Analysis based on the lipid profile of CTRL and RUNX2 KD TPC1 identified by untargeted lipidomics. C) Volcano plots showing altered Hex1Cer, Hex2Cer, and Hex3Cer lipids. D) RUNX2 silencing check by Western Blot analysis on CTRL and RUNX2 KD MDA-MB231. E-F) Resipher live OCR measurement in MDA-MB231. Panel E shows the OCR curves of a representative experiment. The histogram in panel F shows the FC increase of the OCR normalized on cell number. G-H) Seahorse Mitostress test analysis in MDA-MB231. Panel G shows the OCR curves of a representative experiment. Histogram (H) shows the basal, ATP-production linked, and maximum OCR FC increase in MDA-MB1 RUNX2 KD compared to CTRL cells (n=3). I) qRT-PCR showing the FC expression of *RUNX2* and *SREBF1* in RUNX2 KD over CTRL MDA-MB231. Bars represent the Mean±SEM of three independent experiments.

Hex1Cer= Hexosylceramides; Hex2Cer= Dihexosylceramides; Hex3Cer= Trihexosylceramide. *p≤0.05.

Figure S5

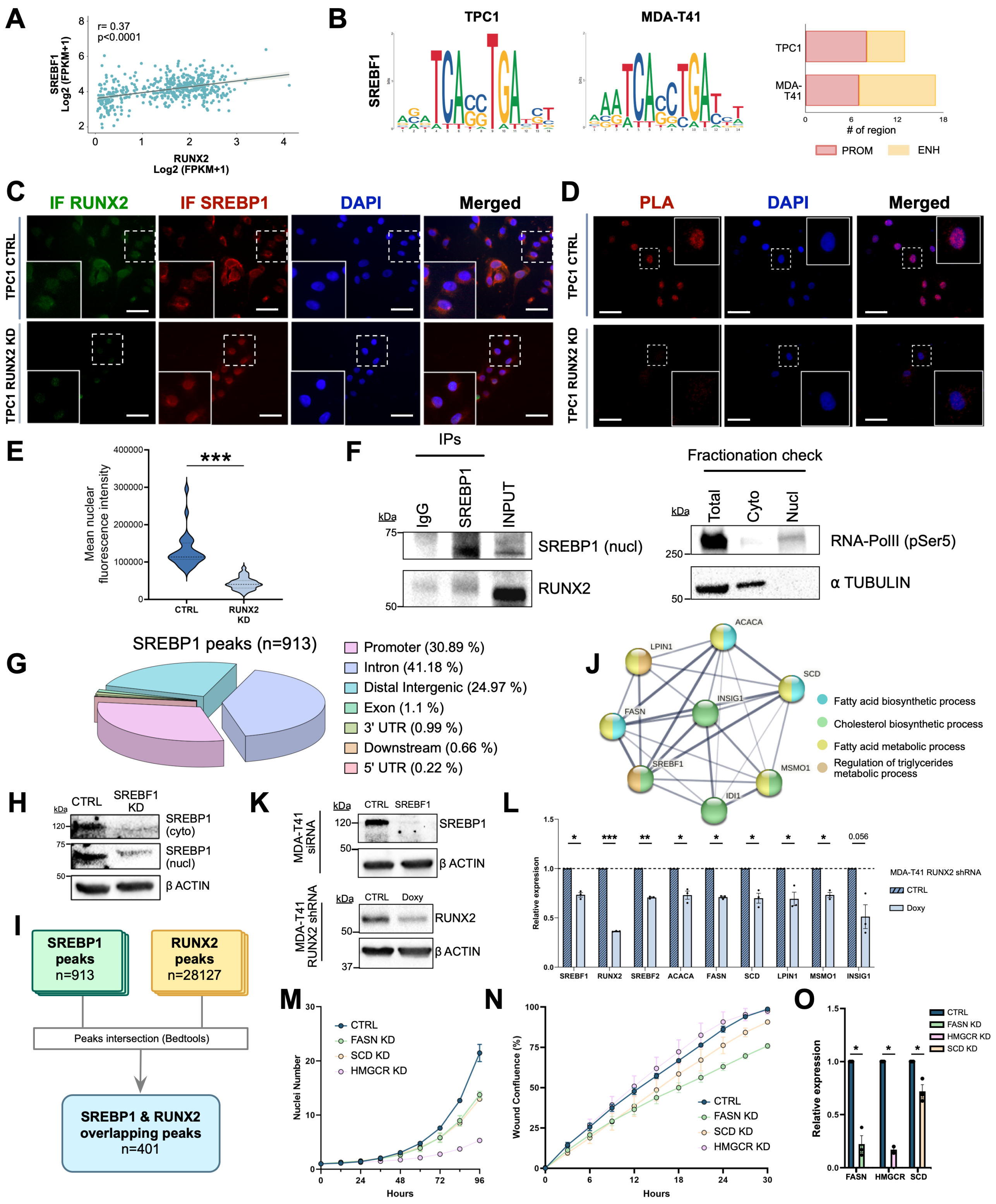

Figure S5. A) Spearman correlation analysis of *RUNX2* and *SREBF1* expression in TCGA-THCA dataset. The line represents linear regression, and the grey area shows the confidence interval. The correlation coefficient (r) and relative p-value (p) are shown on the graphs. B) SRE motif search analysis performed by FIMO on lipogenesis genes' regulatory regions. Sequence logos on the left show the representative SREBP1

motifs identified in TPC1 and MDA-T41. The bar plot on the right shows the number of ChromHMM-identified promoters and ENHs with SRE motifs for each cell line. C-D) RUNX2 and SREBP1 immunofluorescence (A) and PLA (B) in CTRL and RUNX2 KD TPC1. Scale bars = 50 µM. E) Violin plot showing the PLA nuclear signal quantification in CTRL and RUNX2 KD TPC1. ***p<0.0001 F) Co-IP experiment on crosslinked TPC1 nuclei. Western blot on the right shows the fractionation check. G) Pie chart showing the assignment of SREBP1-peaks identified by ChIP-seq in TPC1 to genomic regions. H) SREBP1 silencing checked by Western Blot analysis on CTRL and *SREBF1* KD TPC1. I) Schematic workflow analysis used to identify RUNX2-SREBP1 co-bound genomic regions. J) STRING analysis of representative RUNX2-SREBP1 common target genes. K) Western blot analysis of SREBP1 (upper panel) and RUNX2 (lower panel) knockdown efficiency following RNA-interference. L) qRT-PCR showing the FC expression of lipid metabolism-related genes in *RUNX2* KD (doxy) vs CTRL cells. Bars represent the Mean±SEM of three independent experiments. M-N) Proliferation (M) and migration (N) assays in FASN KD, SCD KD, HMGCR KD, and CTRL TPC1. Curves showed the Mean±SEM of two independent experiments. O) Validation of target gene silencing by qRT-PCR. *p≤0.05.

Figure S6

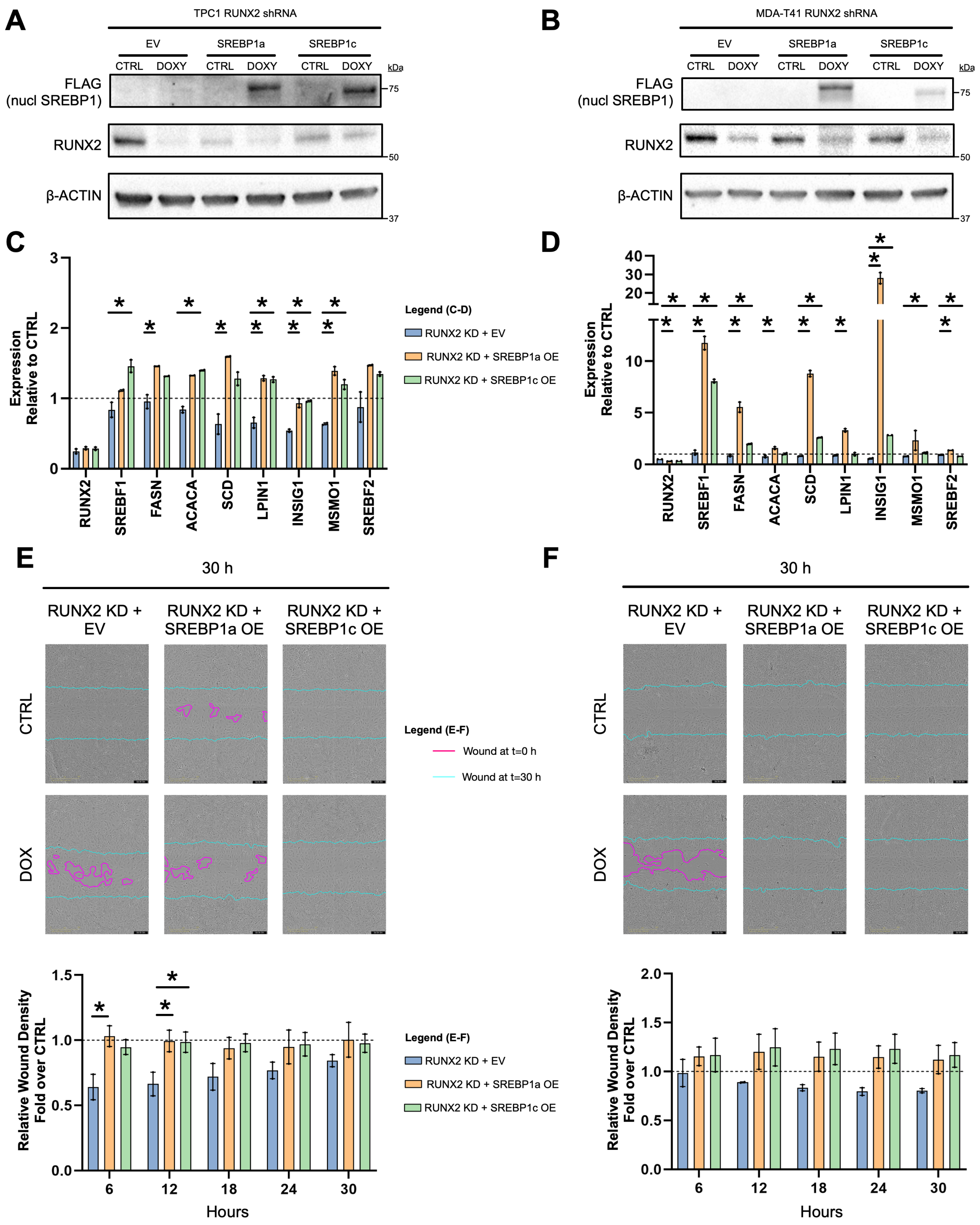

Figure S6. (A-B) Western Blot showing RUNX2 KD and FLAG-SREBP1 overexpression (OE) in TPC1 (A) and MDA-T41 (B). Cells untreated with doxycycline were used as control samples for the induction. Phenotype rescue was assessed by comparing cells co-infected with RUNX2 shRNA and PCW-SREBP1a/c (RUNX2 KD + SREBP1a OE; RUNX2 KD + SREBP1c OE) to those with RUNX2 shRNA and PCW-EV

(RUNX2 KD + EV). (C-D) qRT-PCR analysis showing the expression level of common RUNX2-SREBP1 targets in TPC1 (C) and MDA-T41 (D) cells. Data are shown as FC expression of RUNX2 KD and SREBP1a/c OE relative to the respective control samples. Bars represent the Mean±SEM of two experiments. (E-F) Wound healing assay to assess migration in TPC1 (E) and MDA-T41 (F) upon RUNX2 KD and SREBP1 OE induction. On top are representative images showing the wound area at the experiment endpoint (30 h). On the bottom, histograms showing the FC wound close in RUNX2 KD + EV, SREBP1a OE, and SREBP1c OE cells relative to their respective controls. Bars show the Mean±SEM of two experiments. *$p \leq 0.05$.

Figure S7

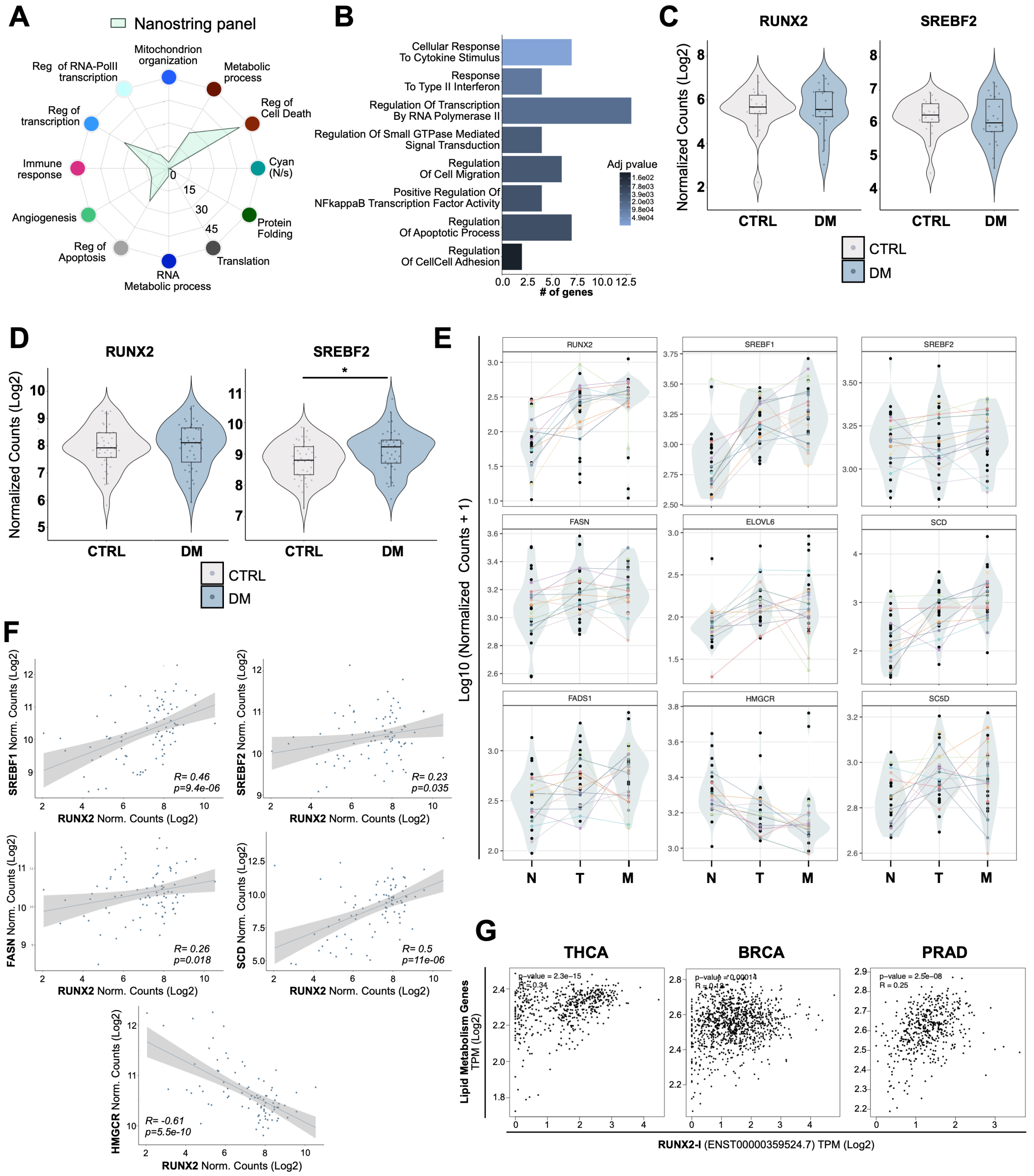

Figure S7. A) Nanostring panel genes selection. Spider plot with percentages of genes included in the Nanostring panel for each RUNX2-dependent disease module that we previously identified (E. Vitale et al., Cancer Communications, 2022). B) GO-BP enrichment analysis of downregulated genes in DM samples compared to control cells. C-D) Violin Plots showing the *SREBF2* and *RUNX2* normalized counts in DM and CTRL PTCs (C) and TNBC (D). E) Violin plot showing *RUNX2*, *SREBF1*, *SREBF2*, and lipid metabolism

enzymes expression in normal, primary tumors, and metastases from patients included in the publicly available cohort. Dots represent the samples. Colored lines link matched samples from the same patients. F) Pearson correlation analysis conducted on the publicly available dataset. The line represents linear regression, and the grey area shows the confidence interval. G) Pearson correlation analysis of RUNX2 expression and a 27-gene lipid metabolism-related signature (identified as RUNX2 targets in TPC1 cells, Fig. 2F) across Thyroid Cancer (THCA), Breast Cancer (BC), and Prostate Cancer (PRAD) datasets from The Cancer Genome Atlas (TCGA).

Supplementary tables

Table S1. ChrHMM results.

	TPC1	MDA-T41	
**1_TssA_PolII**	14.4	15.3	
**2_PromA_PolII**	24.6	11	
**3_PromA**	11	13.3	
**4_PromW**	13.8	13.4	
**5_EnhS_PolII**	34.2	25.8	
**6_EnhS**	55.4	91.2	
**7_EnhW_PolII**	34.9	15	
**8_EnhW**	83.9	112.8	
**9_RNA-PolII**	209.3	57.6	
**10_RNA-PolII_low**	167	218.8	
**11_No_sign**	2438.7	2513.9	
	Mb		

Table S2. TPC1 untargeted metabolomics.

HMDB_Metabolite	HMDB_ID	FC RX vs NT
1-Deoxy-D-xylulose 5-phosphate	HMDB0001213	-1,76
2,3-Diphosphoglyceric acid	HMDB0001294	-5,83
2,3-Diphosphoglyceric acid	HMDB0001294	-5,99
2-Hydroxyglutaric acid	HMDB0000694	-2,56
4-Hydroxycinnamic acid	HMDB0002035	-1,46
Acetylcholine	HMDB0000895	-1,49
Acetylcysteine	HMDB0001890	-5,52
Adenine	HMDB0000034	-1,44
ADP	HMDB0001341	-1,73
alpha-Aminobutyric acid	HMDB0245013	-1,93
Asparagine	HMDB0000168	-4,69
Aspartic acid	HMDB0000191	-2,68
CDP	HMDB0001546	-2,22
Citric acid	HMDB0000094	-2,54
Creatine	HMDB0000064	-2,3
Creatinine	HMDB0000562	-2,22
Cystathionine	HMDB0000099	-2,1
Cytidine monophosphate	HMDB0000095	-1,85
Cytidine triphosphate	HMDB0000082	-2,55
Deoxyadenosine monophosphate	HMDB0000905	-2,77
Dihydrothymine	HMDB0000079	-1,42
Dihydroxyacetone phosphate	HMDB0001473	-1,83
D-Ribose 5-phosphate	HMDB0001548	-2,51
Fructose-1,6-diphosphate	HMDB0244240	-2,92
Gluconic acid	HMDB0000625	1,39
Glutamic acid	HMDB0000148	-2
Glutamine	HMDB0000641	-1,51
Glutathione	HMDB0000125	-1,46
Glycerophosphocholine	HMDB0000086	-1,73
Guanosine diphosphate	HMDB0001201	-2,71
Histidine	HMDB0000177	-1,38
Hypotaurine	HMDB0000965	-2,99
Kynurenine	HMDB0000684	-3,09
Leucine	HMDB0000687	-1,38
Lysine	HMDB0000182	-1,3
Malic acid	HMDB0000156	-4,07
Methionine	HMDB0000696	-1,33
Mevalonic acid	HMDB0000227	-1,89
N(5)-Acetylornithine	HMDB0242109	-2,42
N(6)-Methyllysine	HMDB0002038	-1,42
N-Acetylaspartylglutamic acid	HMDB0001067	-1,6
N-Acetylhistamine	HMDB0013253	-3,55
N-Acetyl-L-aspartic acid	HMDB0000812	-1,87
N-Acetyl-L-glutamic acid	HMDB0001138	-1,56
NAD	HMDB0000902	-2,05

N-methyl-L-glutamic Acid	HMDB0062660	1,23
O-Phosphoethanolamine	HMDB0000224	-1,64
Oxidized glutathione	HMDB0003337	-2,19
Pantothenic acid	HMDB0000210	-2,68
Phosphocreatine	HMDB0001511	-4,52
Phosphogluconic acid	HMDB0256477	-4,06
Phosphoserine	HMDB0000272	-3,33
Proline	HMDB0000162	-2,69
Pyridoxal	HMDB0001545	-1,7
Pyridoxine	HMDB0000239	-1,44
Saccharopine	HMDB0000279	8,69
S-Lactoylglutathione	HMDB0001066	-10,48
Spermidine	HMDB0001257	-2,07
Taurine	HMDB0000251	-2,08
Threonine	HMDB0000167	-1,29
Uridine 2'-phosphate	HMDB0011641	-1,76
Uridine 5'-diphosphate	HMDB0000295	-2,22
Uridine diphosphate-N-acetylglucosamine	HMDB0000290	-3,69
Uridine triphosphate	HMDB0000285	-2,51
Valine	HMDB0000883	-1,4
DG(22:6(4Z,7Z,11E,13Z,15E,19Z)-2OH(10S,17)/0:0/i-16:0)	0	4,02
DG(22:6(4Z,7Z,11E,13Z,15E,19Z)-2OH(10S,17)/0:0/i-17:0)	0	16,08
FAHFA(16:1(9Z)/6-O-16:0)	0	11,26
LysoPE(22:6(4Z,7Z,10Z,13Z,16Z,19Z)/0:0)	0	2,03
LysoSM(d18:0)	HMDB0012082	1,87
PA(18:1(9Z)-O(12,13)/i-21:0)	0	2,94
PA(20:2(11Z,14Z)/18:0)	0	7,12
PA(20:4(5Z,7E,11Z,14Z)-OH(9)/22:6(4Z,7Z,10Z,13Z,16Z,19Z))	0	-1,91
PA(22:6(4Z,7Z,10Z,13Z,16Z,19Z)/15:0)	0	3,04
PA(i-22:0/8:0)	0	-1,63
PC(O-16:0/16:1(9Z))	0	2,44
PE(18:1(9Z)-O(12,13)/P-16:0)	0	2,33
PE(20:5(6E,8Z,11Z,14Z,17Z)-OH(5)/P-16:0)	0	3,42
PE(P-18:0/14:0)	0	-2,97
PE(P-18:1(9Z)/14:1(9Z))	0	2,56
PE-NMe2(18:1(9Z)/14:0)	0	2,9
PE-NMe2(20:5(5Z,8Z,11Z,14Z,17Z)/15:0)	0	8,82
PE-NMe2(22:1(13Z)/15:0)	0	2,97
PG(22:5(7Z,10Z,13Z,16Z,19Z)/18:0)	0	2,76
SM(d18:2(4E,14Z)/16:0)	0	4,73
TG(i-17:0/8:0/8:0)	0	11,95

Table S3. MDA-T41 untargeted metabolomics. Altered metabolites in common with TPC1 are marked in bold.

HMDB_Metabolite	HMDB_ID	RX vs NT
3-Mercaptopyruvic acid	HMDB0001368	6,72
Adenine	**HMDB0000034**	**-1,84**
Adenosine monophosphate	HMDB0000045	-1,79
ADP	**HMDB0001341**	**-2,19**
Allantoin	HMDB0000462	1,48
Arginine	HMDB0000517	1,43
CDP	**HMDB0001546**	**-2,73**
Citric acid	**HMDB0000094**	**-2,47**
Cytidine 3'-monophosphate	HMDB0240312	-1,73
dCDP	HMDB0001245	-4,48
D-Glycerate 3-phosphate	HMDB0060180	-5,16
D-Ribose 1-phosphate	HMDB0250796	-3,82
Fructose 1,6-bisphosphate	HMDB0001058	-3,19
Glutamic acid	**HMDB0000148**	**-1,79**
Glutamine	**HMDB0000641**	**1,92**
Glutathione	**HMDB0000125**	**-4,85**
Glyceraldehyde 3-phosphate	HMDB0001112	-2,23
Glyceric acid	HMDB0000139	-2,44
Guanosine 2'-monophosphate	HMDB0240586	-5,92
Gulonic acid	HMDB0003290	2,33
Hypotaurine	**HMDB0000965**	**-6,37**
Leucine	**HMDB0000687**	**1,78**
Lysine	**HMDB0000182**	**2,1**
Malic acid	**HMDB0000156**	**-3,71**
Methionine	**HMDB0000696**	**1,4**
N-Acetylaspartylglutamic acid	**HMDB0001067**	**-4,61**
N-Acetyl-L-aspartic acid	**HMDB0000812**	**-2,36**
N-Acetyl-L-glutamic acid	**HMDB0001138**	**-4,82**
Oleic acid	HMDB0000207	5,82
Ornithine	HMDB0000214	1,46
Pantothenic acid	**HMDB0000210**	**-2,98**
Phosphoenolpyruvic acid	HMDB0000263	-3,06
Phosphogluconic acid	**HMDB0256477**	**-8,94**
Phosphoserine	**HMDB0000272**	**-4,45**
Serine	HMDB0000187	1,42
Taurine	**HMDB0000251**	**-3,19**
Tyrosine	HMDB0000158	1,34
Uridine diphosphate-N-acetylglucosamine	**HMDB0000290**	**-6,65**
Uracil	HMDB0000300	-1,59
Uric acid	HMDB0000289	1,58
Uridine 2'-phosphate	**HMDB0011641**	**-2,21**
Uridine 5'-diphosphate	HMDB0000295	-2,57
Uridine diphosphate glucose	**HMDB0000286**	**-4,24**

Table S4. Clinical-pathological features of TC patients included in the study.

	**CT n=24**	**DM n=24**	***pValue***
**Age**			0.38
Mean ± sd	53±14	58±19	
na	5	1	
**Sex**			0.21
M	5 (21%)	10 (42%)	
F	19 (79%)	14 (58%)	
**pN**			<0.001
0	24 (100%)	4 (17%)	
1	-	19 (83%)	
na	-	1	
**pT**			<0.001
1	16 (67%)	1 (4%)	
2	5 (21%)	2 (9%)	
3	3 (12%)	15 (65%)	
4	-	5 (22%)	
na	-	1	
**pSTAGE**			<0.001
1	10 (59%)	2 (9%)	
2	4 (23%)	4 (18%)	
3	3 (18%)	4 (18%)	
4	0	12 (55%)	
na	7	2	

Table S5. Clinical-pathological features of BC patients included in the study.

	CT naive (n=34)	CT post treatment (n=6)	DM naive (n=25)	DM post treatment (n=14)
**Age**				
Mean ± sd	52±13	53±7	63±14	51±11
**pN**				
0	28 (82%)	3 (50%)	5 (22%)	7 (50%)
1	6 (18%)	3 (50%)	8 (35%)	3 (21.5%)
2	-	-	3 (13%)	3 (21.5%)
3	-	-	4 (17%)	-
X	-	-	5 (22%)	1 (7%)
**pT**				
0	-	-	-	1 (7%)
1	26 (76%)	5 (83%)	7 (35%)	6 (43%)
2	7 (21%)	-	11 (55%)	7 (50%)
3	1 (3%)	1 (17%)	2 (10%)	-
na	-	-	5	-
**pSTAGE**				
1	23 (68%)	2 (33%)	-	-
2	9 (26%)	4 (67%)	-	-
3	2 (6%)	-	-	-
4	-	-	25 (100%)	14 (100%)

Table S6. List of sgRNAs and shRNAs.

Name	Target	Sequence
**RUNX2 sgRNA**	RUNX2-P2 promoter	AATGAGCGACGTGAGCCCGG
**SREBF1 sgRNA**	SREBF1 TSS	TCCCTAGGAAGGGCCGTACG
**FASN sgRNA**	FASN TSS	GGTTTAAATAGCGTCGGCGC
**HMGCR sgRNA**	HMGCR TSS	CAGTTAACGCAGTCGCGGAG
**SCD sgRNA**	SCD TSS	TGTAAACTCCGGCTCGTCAT
**NT sgRNA**	-	CTGAAAAAGGAAGGAGTTGA
**RUNX2 shRNA**	RUNX2 CDS	CAGCACTCCATATCTCTACTA

Table S7. List of primers for gene expression.

Target	Forward primer	Reverse primer
**ACACA**	AGTGGGTCACCCCATTGTT	TTCTAACAGGAGCTGGAGCC
**ACTB**	ACCTTCTACAATGAGCTGCG	CCTGGATAGCAACGTACATGG
**FASN**	CTGCTGAGCACAGACGAGAG	GGCCTATCTGGATGGCAGTC
**FDPS**	CGAGAAGGAGCACGCCAATG	GCCAATTTTGCCGGTCACAC
**HMGCR**	GGAACCTCGGCCTAATGAAG	CACCACGCTCATGAGTTTCC
**INSIG1**	CTCTCGGCCAGGAAGCG	CAGCTCCAGAAGTGGTCGTG
**LPIN1**	TGCTGGAGAGCAGCAGAACTC	TAGGGTATGAGGCTGACTGAG
**MSMO1**	ATCATGAGTTTCAGGCTCCATT	AAGCACGATTCCAATGAAAAAT
**PPIA**	GACCCAACACAAATGGTTCC	TTTCACTTTGCCAAACACCA
**RUNX2**	GCTCTTCTTACTGAGAGTGGAAGG	GTGCCTAGGCGCATTTCA
**SC5D**	TGCACTGTTCTTGCTGGAGA	TGAAGGCCTCTGTGAATCCAG
**SREBF1**	GCAAGGCCATCGACTACATT	GGTCAGTGTGTCCTCCACCT
**SREBF2**	CTGAGGCAGGCTTTGAAGAC	GATCTCAACCAGTGTGCTCG

Table S8. List of primers for ChIP.

Target	Forward primer	Reverse primer
**FASN prom**	TCTCTCTGGCTCCCTCTAGG	GACGCTCATTGGCCTGGG
**MSMO1 prom**	TATCCTCACTCCATCCGCGG	CCGTCCTGTCGTTATTGGCC
**Neg CTRL**	GGCTCCGTTGGCTATGCATA	GGGCTCTCTCTCTGATTGGC
**SCD prom**	GGCAGAGGGAACAGCAGATT	GGCTTCTGTAAACTCCGGCT
**SREBF1 prom**	TACAGAGTGTTCAGTGCGGT	GCTCCACGTGCCTCAATTTA
**SREBF1c TSS**	TTGGGGTTACTAGCGGACGT	GTGTGTTGGGCCAGGACTTC
**SREBF2 TSS**	TAACCCTTCACTCCCAGGCC	CCGTCCGGTCATCATCTTAACC

Table S9. List of antibodies.

Name	Code	Manufacturer	*Experimental condition*		
			**Western Blot (dilution)**	**ChIP/IP (µg)**	**IF/PLA (dilution)**
ACACA	C83B10	Cell signaling technology	1/1000	-	-
FASN	C20G5	Cell signaling technology	1/1000	-	-
FLAG	M2	Sigma Aldrich	1/1000	-	-
H3K27ac	ab4729	Abcam	-	2	-
H3K4me1	D1A9	Cell signaling technology	-	1	-
H3K4me3	ab8580	Abcam	-	1	-
Normal Rabbit IgG	2729	Cell signaling technology	-	0.5 to 5	-
RNA-POLII (Rbp1)	D8L4Y	Cell signaling technology	-	5	-
RNA-POLII Phospho S5	D9N5I	Cell signaling technology	1/1000	-	-
RUNX2	D1L7F	Cell Signaling Technology	1/1000	5	-
RUNX2	sc-390351	Santa Cruz Biotechnology	1/2000	-	1/100
SREBF1	14088-1-AP	Proteintech	1/1000	4	1/200
SREBF1	MA5-11685	Thermo Fischer Sc.	1/1000	-	-
SREBF1	E9F4O	Cell signaling technology	-	0.5	-
TOM20	ab78547	Abcam	-	-	1/200
α-TUBULIN	sc-8035	Santa Cruz Biotechnology	1/1000	-	-
β-ACTIN	A1978	Sigma Aldrich	1/5000	-	-
